# Effects of Bariatric Surgery-Related Weight Loss on the Characteristics, Metabolism, and Immunomodulation of Adipose Stromal/Stem Cells in a Follow-Up Study

**DOI:** 10.1155/sci/1212255

**Published:** 2025-05-13

**Authors:** Amna Adnan, Miia Juntunen, Tuula Tyrväinen, Minna Kelloniemi, Laura Kummola, Reija Autio, Mimmi Patrikoski, Susanna Miettinen

**Affiliations:** ^1^Adult Stem Cell Group, Faculty of Medicine and Health Technology, Tampere University, Tampere, Finland; ^2^Tays Research Services, Tampere University Hospital, Wellbeing Services County of Pirkanmaa, Tampere, Finland; ^3^Department of Gastroenterology and Alimentary Tract Surgery, Tampere University Hospital, Wellbeing Services County of Pirkanmaa, Tampere, Finland; ^4^Department of Plastic and Reconstructive Surgery, Tampere University Hospital, Wellbeing Services County of Pirkanmaa, Tampere, Finland; ^5^Biodiversity Interventions for Well-being, Faculty of Medicine and Health Technology, Tampere University, Tampere, Finland; ^6^Faculty of Social Sciences, Tampere University, Tampere, Finland; ^7^Obesity Research Unit, Research Program for Clinical and Molecular Metabolism, Faculty of Medicine, University of Helsinki, Helsinki, Finland; ^8^Finnish Red Cross Blood Service, Advanced Cell Therapy Centre, Helsinki, Finland

**Keywords:** adipose stromal/stem cells, bariatric surgery, inflammation, macrophages, obesity, weight loss

## Abstract

**Background:** The success of adipose stromal/stem cell (ASC)-based therapies may depend on donor characteristics such as body mass index (BMI). A high BMI may negatively impact the therapeutic potential of ASCs, but the effects of weight loss on ASC-mediated immunoregulation have not been extensively studied.

**Methods:** ASCs were obtained from donors with obesity (obASCs) undergoing bariatric surgery and from the same donors after weight loss (wlASCs). Plasma samples, adipose tissue histology, and ASC characteristics, such as mitochondrial respiration and inflammatory factors, were studied before and after weight loss. The immunomodulatory capacity of ob/wlASCs was evaluated in cocultures with prepolarized and preactivated proinflammatory (M1) and anti-inflammatory (M2) macrophages by determining macrophage surface markers, gene expression, and cytokine secretion.

**Results:** Weight loss significantly decreased plasma leptin levels and increased adiponectin levels. After weight loss, crown-like structures (CLSs) were undetectable, and the adipocyte size decreased. Weight loss significantly improved mitochondrial respiration in ASCs and resulted in a notable increase in their proliferative capacity. The proinflammatory marker genes tumor necrosis factor alpha (*TNF-α*), chemokine ligand 5 (*CCL5*), and cyclooxygenase-2 (*COX2*), as well as the proinflammatory cytokine interleukin 12p70 (IL-12p70), were significantly downregulated, while the anti-inflammatory gene tumor necrosis factor-inducible gene 6 (*TSG6*) was also significantly downregulated in ASC monocultures after weight loss. Following weight loss, ASCs exhibited increased proinflammatory properties when cocultured with macrophages, characterized by the downregulation of anti-inflammatory factors, along with the upregulation of several proinflammatory factors, compared with the effects of macrophage monocultures. Conversely, wlASCs demonstrated improved immunosuppressive functions in coculture with macrophages, as indicated by the upregulation of *TSG6* gene expression and interleukin 4 (IL-4) secretion.

**Conclusions:** Weight loss improved donors' metabolic health and partially recovered ASCs' anti-inflammatory gene expression and cytokine secretion profiles in monocultures. However, it was inadequate to fully restore the immunosuppressive functions of ASCs in cocultures with macrophages. Therefore, not only donor BMI but also weight loss history, among other donor characteristics, might be considered for optimal ASC-based therapy.

## 1. Introduction

The potential of adipose stromal/stem cell (ASC)-based therapies relies on their proliferation and differentiation capacity [1–3] as well as their secretory factors affecting angiogenesis, wound healing [4], cell survival, fibrosis, and neovascularization [5]. In particular, the immunomodulatory functions of ASCs have drawn attention to their utilization in cell-based therapies [6–8]. It has been demonstrated that ASCs secrete cytokines and exosomes that regulate the number and activity of immune cells [9, 10]. For example, ASCs can polarize proinflammatory (M1) macrophages toward anti-inflammatory (M2) macrophages [11–13] and modulate T-cell functions [14–16] in an inflammatory milieu.

Obesity-induced chronic low-grade inflammation impacts the physiology of adipose tissue [17] and disturbs the balance and functions of immune cells, such as T cells and macrophages, residing in adipose tissue [18]. M1 and M2 macrophages are abundant in adipose tissue, and their phenotypes are regulated by the surrounding environment [19]. In nonobese individuals, M2 macrophages maintain adipose tissue homeostasis, preventing inflammation and promoting insulin sensitivity [20], whereas M1 macrophages are associated with obesity-induced inflammation [21].

Obesity-induced inflammation not only interrupts the macrophage equilibrium in adipose tissue [22, 23] and accelerates the secretion of proinflammatory adipokines and cytokines [24] but also may affect ASC functions [25], such as their immunomodulatory capacity [16, 25, 26]. Compared with ASCs derived from normal-weight donors, human ASCs derived from obese donors (obASCs) exerted reduced immunosuppressive effects on mouse T cells in vitro [27], with similar effects on mouse macrophages in vivo [8, 28, 29]. In contrast, compared with obASCs, ASCs derived from donors of normal weight exerted strong immunosuppressive effects on the macrophage phenotype [30]. Additionally, a recently published study showed that ASCs derived from previously morbidly obese donors behaved similarly to ASCs derived from overweight donors, as both promote M2 macrophage polarization and exhibit a mixed pro- and anti-inflammatory secretome [31].

Bariatric surgery is an effective weight loss procedure [32] that may restore metabolic functions by reducing subcutaneous fatty acid uptake and positively affecting hepatokines and plasma inflammatory markers [22, 23]. While the majority of studies focusing on the effects of donor weight on ASC properties have compared ASCs derived from donors with either normal weight or obesity [8, 16, 25–30], only a few studies have focused on the effects of weight loss [31, 33–35]. Moreover, only a few studies have reported the effects of bariatric surgery-associated weight loss, including reduced DNA damage, improved viability, and decreased adipogenic differentiation of either ASCs or the stromal vascular fraction [33, 34]. Additionally, some studies have shown the effects of obesity, weight loss, and bariatric surgery-related weight loss on angiogenic [36] and pericyte functions [35]. However, some of these studies have not mentioned the weight loss methods employed [36]. Few studies have examined the clinical implications of weight loss-induced changes in ASCs despite their use in regenerative medicine and immunotherapy [31, 33–35]. For instance, Alves et al. reported that ASCs from ex-obese donors exhibit an altered immunomodulatory profile, polarizing M0 macrophages toward an M2-like phenotype with a mixed (M1/M2) secretory response. This suggests that weight loss history could influence ASC-based therapies for inflammatory diseases such as Crohn's disease and spinal cord injury [31]. Similarly, Schmitz et al. [34] observed a negative correlation between donor weight loss history and ASC functional activities, including proliferation and migration capacity, indicating that ASCs from individuals who have lost weight may have diminished regenerative potential. In addition, Silva et al. found that weight loss following bariatric surgery alters ASC properties and increases pericyte function, monocyte chemoattractant protein-1 (MCP-1) secretion, and lipid accumulation, which could affect their regenerative potential. However, further molecular investigations are needed to ensure that autologous ASC therapies remain effective post-weight loss [35]. Despite these findings, there is a lack of follow-up in vitro and in vivo studies from the same individual before and after the weight loss investigating the long-term effects of bariatric surgery-induced weight loss on ASC function, particularly their immunomodulatory interactions with macrophages, which are key immune cells regulating adipose tissue homeostasis [37]. Understanding these changes is critical to optimizing ASC-based therapeutic applications and ensuring their efficacy in clinical settings. Therefore, this study aimed to analyze the effects of weight loss on the characteristics and immunomodulatory functions of ASCs derived from the same donors before and after bariatric surgery-associated weight loss.

In this study, ASCs were isolated before (obASCs) and after weight loss (wlASCs) from donors who underwent bariatric surgery, maintaining a similar genetic background before and after weight loss. Donor inflammatory status was examined by analyzing plasma C-reactive protein (CRP), leptin, and adiponectin concentrations. From adipose tissue histological samples, adipocyte size and the presence of crown-like structures (CLSs) were evaluated. The surface marker expression and differentiation capacity of obASCs and wlASCs were also analyzed. Moreover, the oxygen consumption rate (OCR) of ASCs was studied by examining mitochondrial functions. To evaluate the immunomodulatory capacity of ASCs, macrophages were polarized and activated toward the M1 and M2 phenotypes from peripheral blood mononuclear cells (PBMCs) and studied in direct cocultures with ASCs to mimic the environment of adipose tissue where cells are in contact with each other [22, 23]. Surface marker profiles, pro- and anti-inflammatory gene expression, and cytokine secretion in mono- and cocultures were assessed to study cell‒cell interactions.

## 2. Materials and Methods

### 2.1. Subjects

Subcutaneous adipose tissue samples were obtained from donors who underwent bariatric surgery (*n* = 6, body mass index [BMI] 39.89–55.02, age 39–53) at the Tampere University Hospital (TAUH), Department of Gastroenterology and Alimentary Tract Surgery, and from the same donors (*n* = 6, BMI 25.18–31.46, age 41–56) after weight loss at the TAUH, Department of Plastic and Reconstructive Surgery. The samples were obtained with the donors' written informed consent and processed in accordance with supportive statements by the Ethics Committee of the Expert Responsibility area of TAUH (R16036). The donor characteristics are summarized in Table S1. The study was conducted in accordance with the revised Declaration of Helsinki 1975.

### 2.2. Plasma Inflammatory Markers

The levels of plasma CRP, leptin, and adiponectin were measured before (*n* = 6) and after weight loss (*n* = 6) by enzyme-linked immunosorbent assays (ELISAs; R&D Systems) following the manufacturer's instructions.

### 2.3. Adipose Tissue Histology

The adipose tissue samples before (*n* = 6) and after weight loss (*n* = 6) were fixed and stabilized with PAXgene tissue fix and PAXgene tissue stabilizer (PAXgene Tissue System, PreAnalytix GmbH) and stored at −20°C. The adipose tissue was sectioned (7 µm) and stained with hematoxylin and eosin (HE). Adipose tissue chromogenic staining was performed using a mouse monoclonal antibody against CD68 (M087601-2, 1:100 dilution; Dako Agilent Technologies) with a Ventana BenchMark GX with an Ultraview Dab Kit (Roche Diagnostics) and an Autostainer 480S (Lab Vision Corporation). The histological images were taken with an Olympus VS200 microscope scanner (Olympus Life Science), and adipocyte size was calculated with Fiji-ImageJ software [38]. To record adipocyte area, random sections of the HE images were selected from which single adipocytes were manually marked, and their areas were calculated using the ROI Manager tool in ImageJ. Only complete adipocytes in the field of view were considered.

### 2.4. Cell Isolation and Culture

Human ASCs were isolated from subcutaneous adipose tissue before (obASCs) and after weight loss (wlASCs), cultured, and expanded as described previously [16, 39] in alpha MEM (Thermo Scientific) supplemented with human serum (Serana) and 1% P/S (Lonza) (basic medium, BM). The experiments with ASCs were conducted in passages 4–5.

To assess the effect of inflammation on the immunomodulatory activity of ASCs, ASCs were cultured in RPMI-1640 supplemented with L-glutamine, sodium bicarbonate (SIGMA, Life Science), recombinant human interferon-gamma (IFN*ɣ*) (10 ng/mL; R&D Systems), 10% fetal bovine serum (FBS; Thermo Fisher Scientific, Gibco) and 1% P/S for 48 h prior to coculture with polarized and activated macrophages.

Allogenic human PBMCs (Finnish Red Cross Blood Service Helsinki) from healthy donors were isolated from buffy coat samples with Ficoll Paque PLUS (density of 1.077 g/mL; GE Healthcare). The isolated cells were cryopreserved in the nitrogen gas phase.

### 2.5. Assessment of Mitochondrial Respiration

Mitochondrial respiration levels in obASCs (*n* = 6) and wlASCs (*n* = 6) were assessed using a Cell Mito Stress Test Kit (Agilent Technologies, Inc.). Briefly, a total of 25,000 cells/well were seeded in a 24-well plate for 48 h. On the day of analysis, the maintenance medium (10% FBS in RPMI) was replaced with a Seahorse assay medium supplemented with 10 mM glucose, 1 mM pyruvate, and 2 mM glutamine. The OCR was measured at the basal level and after the addition of oxidative phosphorylation modulators; this parameter included proton leakage after oligomycin treatment (1 μM), the maximal OCR after carbonyl cyanide-p-trifluoromethoxyphenylhydrazone treatment (2 μM), and nonmitochondrial respiration after treatment with antimycin A and rotenone (0.5 μM). Measurements were taken with an XFe24 Seahorse Analyzer, and the data were analyzed with an Agilent Seahorse Analytics data acquisition device (Agilent Technologies). A line graph for mitochondrial respiration was created based on the kinetic profile of the 120-min measurement period from all donors.

### 2.6. Surface Marker Profile of ASCs

For surface marker analyses, ASCs were cultured in BM. To verify the phenotypic characteristics of the ASCs according to the International Society for Cell & Gene Therapy (ISCT) [40] and the International Federation for Adipose Therapeutics and Science (IFATS) [41], the surface markers CD13-BV421, CD14-APC, CD19-APC, CD29-FITC, CD31-BV421, CD34-APC, CD36-APC, CD44-FITC, CD45RO-APC, CD54-FITC, CD73-PE, CD90-APC, CD105-PE, CD146-BV421, CD235A-BV421 and human leukocyte antigen–DR isotype (HLA-DR) BV421 (Table S2) were assessed using a CytoFlex S flow cytometer (Beckman Coulter). A total of 10,000 events were recorded during the flow cytometry analysis. The percentage and median fluorescence intensity (MFI) of each positive cell were determined, and the data were analyzed using FlowJo software (BD Biosciences).

### 2.7. Proliferation and Multipotent Differentiation Capacity

The proliferation rates of obASCs (*n* = 5) and wlASCs (*n* = 5) were analyzed with a Cell Counting Kit-8 (CCK-8) assay (Dojindo Laboratories) according to the manufacturer's instructions. To evaluate the multipotential differentiation capacity of ASCs, obASCs (*n* = 5) and wlASCs (*n* = 5) were differentiated into adipogenic, osteogenic, and chondrogenic lineages [16]. For more details about the proliferation assay and multipotential differentiation, see Supporting Information 1.

### 2.8. Macrophage Polarization, Activation, and Coculture Assay

M1 and M2 cells were polarized from PBMCs [42]. A total of 2 million PBMCs in basal RPMI-1640 medium were seeded in 12-well plates (Nunc, Thermo Fisher Scientific) and incubated for 2 h at 37°C. Afterward, a polarization medium was added to each well (Table S3), and the cells were incubated for 6 days at 37°C. On Day 6, the polarization medium was replaced with the activation medium (Table S3). For cocultures, on Day 7, 80,000 obASCs and wlASCs in M1/M2 activation medium were added to representative coculture wells and incubated at 37°C for 3 days. On Day 10, mono- and cocultures were detached with Accutase cell dissociation reagent (Thermo Fisher Scientific StemPro) with the help of a cell scrapper (USA Scientific, Inc.) for subsequent assays. A graphical illustration of the assay timeline is provided in Figure S1. On Day 11, total messenger RNA (mRNA) and conditioned media samples were collected to analyze the gene expression and cytokine secretion of macrophages and monocultured and cocultured ASCs, which were subsequently stored at −80°C. Additionally, monocultures of obASCs (*n* = 4) and wlASCs (*n* = 4) were cultured in RPMI-1640 supplemented with L-glutamine and sodium bicarbonate with 10% FBS and 1% P/S supplemented with IFN*ɣ*.

### 2.9. Surface Markers of Macrophages

The immunophenotypes of M1 and M2 monocultures (Figures S10 and S11) and their coculture with obASCs and wlASCs were studied by multicolor flow cytometric analysis in a FACSAria (BD Biosciences) and CytoFlex S. Monoclonal antibodies against CD86-PE, HLA-DR-FITC, CD163-CF594, CD90-APC, CD206-BV421, and CD11C-PECy7 were used (Table S2). FACS analysis was performed with BD CompBead Plus beads (BD Bioscience), and cell viability was determined with Fixable Viability Stain 510 (BD Biosciences). M1 and M2 type macrophages were selected for the analysis based on their size and granularity (Figures S10 and S11). The obASCs and wlASCs were excluded from the macrophage cocultures based on CD90 expression (Figure S12B,E) to analyze the live population of M1 and M2 macrophages (Figure S12C,F). A total of 20,000 events were recorded during flow cytometry analysis. The raw data were analyzed using FlowJo software. MFI (*n* = 8) and percentage (*n* = 10) data for a population of positive cells were analyzed (Supporting Information 1).

### 2.10. Gene Expression Analysis

Total mRNA was isolated from mono- and cocultures of obASCs (*n* = 4), wlASCs (*n* = 4), and macrophages using a NucleoSpin RNA II kit (Macherey-Nagel GmbH & Co.). Complementary DNA was synthesized with a High-Capacity cDNA Reverse Transcription Kit (Applied Biosystems), and quantitative real-time reverse transcription polymerase chain reaction (qRT-PCR) was performed with a QuantStudio 12K Flex System (Applied Biosystems) using TaqMan Fast Advanced Master Mix (Thermo Fisher) and Custom TaqMan Array Plates (Applied Biosystems) (Table S4). 18S ribosomal RNA and glyceraldehyde 3-phosphate dehydrogenase were used as reference genes for normalizing the qRT-PCR data. Relative quantification of gene expression was performed using the 2^−*ΔΔ*Ct^ method [43]. In ASC monocultures, the gene expression levels are shown relative to the average expression in obASCs, whereas, in cocultures, the gene expression levels are shown relative to the average expression in macrophage monocultures.

### 2.11. Cytokine Analysis

The cytokines produced by mono- and cocultures of obASCs (*n* = 4), wlASCs (*n* = 4), and macrophages during the macrophage polarization and activation assays were studied in conditioned media. Eleven cytokines and chemokines (interleukin 1 receptor antagonist [IL-1RA], thymus- and activation-regulated chemokine [TARC], macrophage inflammatory protein 1 alpha [MIP-1*α*], macrophage-derived chemokine [MDC], Monocyte chemoattractant protein-1 [MCP-1], interleukin 1 beta [IL-1*β*], interleukin 4 [IL-4], interleukin 6 [IL-6], interleukin 10 [IL-10], interleukin 12p70 [IL-12p70] and tumor necrosis factor alpha [TNF-*α*]) were assessed with a custom-made V-PLEX Assay (Mesoscale Diagnostics, LLC) [44] (Table S5). All the assays were performed in duplicate. Analyses were performed using an MSD QuickPlex SQ120 instrument and DISCOVERY WORKBENCH (MesoScale Diagnostics, LLC).

### 2.12. Statistical Analysis

Statistical analysis was performed using GraphPad Prism software version 9.0.0 (GraphPad, Inc.). The Shapiro–Wilk test was used to assess the normality of the data. Based on its results, appropriate parametric or nonparametric tests were selected. The data are presented as the mean and standard deviation for flow cytometric analysis and as the minimum to maximum with boxplots for all other analyses. The Wilcoxon signed-rank test was used to analyze plasma inflammatory marker and ASC gene expression in monocultures, while paired sample *t*-tests were used to analyze adipocyte area, ASC surface marker expression, ASC proliferation, mitochondrial respiration data, and ASC cytokine secretion in monocultures and macrophage monocultures. A paired sample *t*-test was used for normally distributed data, whereas the Wilcoxon signed-rank test was used for nonnormally distributed data. Additionally, repeated measures one-way ANOVA was used to calculate the *p* values for macrophage and ASC mono- and coculture surface marker expression, gene expression and cytokine analysis. A line graph for mitochondrial respiration was created based on the kinetic profile of the 120-min measurement period from all donors. Additionally, *p* ≤ 0.05 indicated statistical significance.

## 3. Results

### 3.1. Decreased Plasma Levels of Inflammatory Markers After Weight Loss

Bariatric surgery has been demonstrated to facilitate weight loss and improve metabolic functions [32, 45–48]. In our study, we observed that the BMI of ASC donors (*n* = 6) significantly decreased after bariatric surgery (ΔBMI: average 15.41, *p*=0.0313) (Table S6).

**Figure 1:**
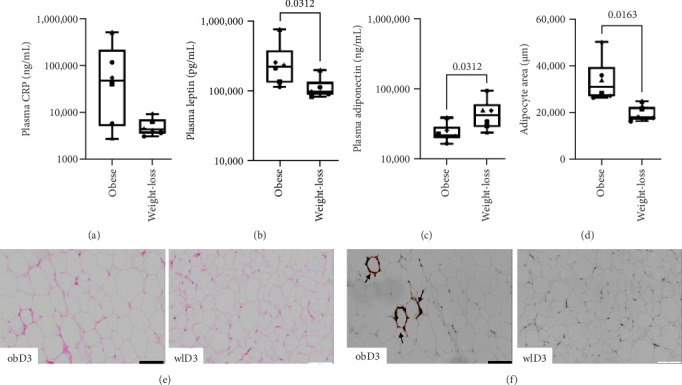
Effects of weight loss on plasma inflammatory (CRP, leptin) and anti-inflammatory (adiponectin) marker levels, adipocyte areas, and crown-like structures (CLSs). Decreased plasma CRP and leptin levels and increased adiponectin levels were detected after weight loss; *n* = 6. The Wilcoxon test for paired samples was used. *p* Values < 0.05 were considered significant. (A) CRP, (B) leptin, and (C) adiponectin levels. Hematoxylin and eosin (HE)-stained histological samples of AT after weight loss presented a decreased adipocyte area and a lack of CLS following CD68 chromogenic staining. (D) Adipocyte area in µm before and after weight loss. (E) Representative HE histological samples before and after weight loss from one donor. (F) Representative CD68-positive chromogenic histological samples before and after weight loss from one donor. Arrows indicate the accumulation of CLS around adipocytes. CRP, C-reactive protein; obD, donor before weight loss; wlD, donor after weight loss. See also Figure S2.

Previous reports have highlighted an imbalance of plasma pro- and anti-inflammatory markers associated with obesity and related metabolic disorders [49, 50]. To determine how weight loss impacts donor inflammatory status, we analyzed the adipokines leptin and adiponectin, as well as CRP, in blood plasma samples from donors before (*n* = 6) and after weight loss (*n* = 6). Our results aligned with those of previous studies showing improvements in plasma adipokines [24, 51] and CRP levels [52, 53] after weight loss. Our findings revealed a notable decrease in the inflammatory CRP level (Figure 1A), a significant decrease in the leptin level (*p*=0.0312; Figure 1B), and a significant increase in the anti-inflammatory adiponectin level (*p*=0.0312) following weight loss (Figure 1C).

### 3.2. Decreased Adipocyte Size and the Absence of CLSs in Adipose Tissue Samples After Weight Loss

Heinonen et al. [54] demonstrated that obesity-related remodeling of adipose tissue results in adipocyte hypertrophy, which is further associated with the accumulation of M1 macrophages, the formation of CLSs and, ultimately, adipocyte death. To evaluate the impact of weight loss on adipocyte size and the presence of CLSs, we examined adipose tissue sections before (*n* = 6) and after weight loss (*n* = 6). Notably, we observed a significant reduction in the adipocyte area (*p*=0.0163) following weight loss (Figure 1D), which was consistent with previous findings [55–57]. Additionally, our observations revealed dying adipocytes surrounded by CD68^+^ macrophages in adipose tissue samples obtained before weight loss. No CLSs were observed after weight loss (Figure 1F/Figure S2B), supporting the findings of a previous report by Maliniak et al. [58].

### 3.3. Improved Mitochondrial Respiration Capacity of ASCs After Weight Loss

**Figure 2:**
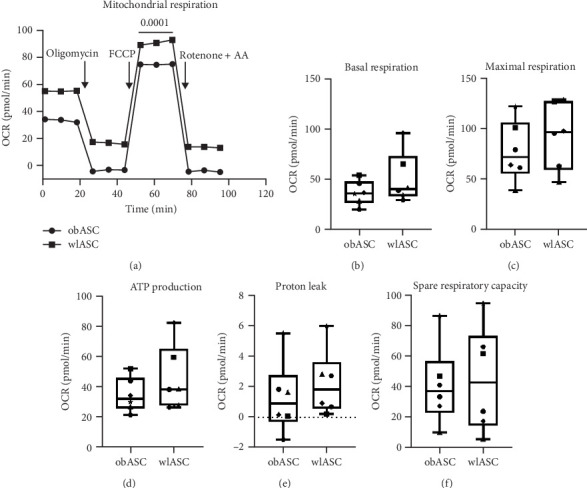
Improved oxygen consumption rates of ASCs after weight loss; *n* = 6. (A) Mitochondrial respiration, (B) basal respiration, (C) maximal respiration, (D) ATP production, (E) proton leakage, and (F) spare respiratory capacity. Paired *t*-test. *p* Values < 0.05 were considered significant. Data are presented as the medians for mitochondrial respiration and as the minimum to maximum values for other measured individual factors. obASCs, ASCs obtained before weight loss; OCR, oxygen consumption rate; wlASCs, ASCs obtained after weight loss.

Obesity-induced inflammation has been demonstrated to induce oxidative stress in adipocytes. This stress impairs their mitochondrial functions and reduces the OCR of ASCs, especially compared to ASCs derived from individuals of normal weight [59–61]. However, bariatric surgery-related weight loss has been shown to increase mitochondrial respiration within adipose tissue [62]. To investigate this phenomenon further, we examined the overall mitochondrial respiration rates of obASCs (*n* = 6) and wlASCs (*n* = 6) by measuring the basal, maximal respiration, ATP production, proton leakage, and spare respiratory capacity of the cells. Although we did not observe significant differences when comparing individual factors (Figure 2), consistent with previous findings, we found that the overall mitochondrial respiration rate over the 120-min measurement period was significantly greater in wlASCs (*p*  < 0.0001) than in obASCs (Figure 2A).

### 3.4. Decreased Adipocyte and Pericyte Surface Marker Levels After Weight Loss

Obesity-induced inflammation may alter the typical surface marker profile of ASCs [16, 63, 64]. In this study, both obASCs (*n* = 5) and wlASCs (*n* = 6) met the defined surface marker criteria established by the ISCT/IFATS for mesenchymal stem cells (MSCs), albeit with minor differences. The cells were positive (>85%) for CD90, CD73, CD105, CD13, CD44, and CD29 and negative (<2%) for CD14, CD45RO, CD235a, HLA-DR, and CD34 (Figures S3 and S4).

Interestingly, the median percentage of CD36-positive cells was significantly lower in wlASCs (*p*=0.0110) than in obASCs (Figure S3F). CD36 serves as a marker for human adipocyte progenitors and mononuclear phagocytes, and its expression is upregulated during obesity and associated with increased adipogenesis [65, 66]. In line with the downregulation of CD36 and the findings of our previous study [63], we observed a reduced adipogenic capacity in wlASCs compared with obASCs.

In contrast to that of CD36-positive cells, the percentage of vascular differentiation marker CD31-positive cells [67] was significantly greater in wlASCs (*p*=0.0456) than in obASCs (Figure S3N). Furthermore, the MFI of the pericytic marker CD146 [68] decreased significantly in ASCs after weight loss (*p*=0.0017) (Figure S4M).

### 3.5. Pro- and Anti-Inflammatory Gene Expression and Cytokine Secretion in ASC Monocultures

#### 3.5.1. Downregulation of Both Pro- and Anti-Inflammatory Gene Expression in ASCs After Weight Loss

**Figure 3:**
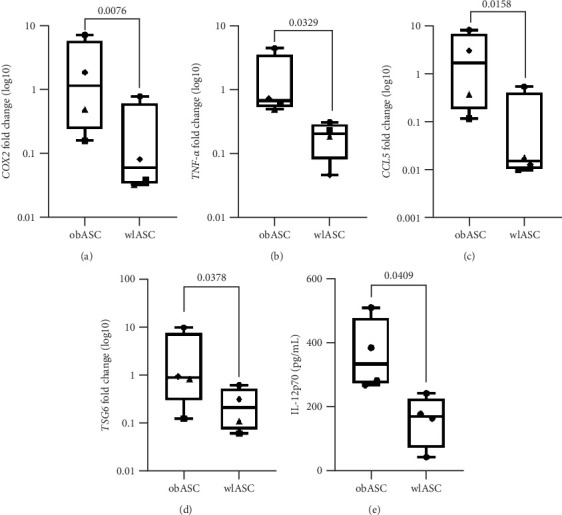
Comparison of gene expression and cytokine secretion between obASCs and wlASCs; *n* = 4. (A)–(D) Gene expression. Box plots of the fold change values and statistical analysis of the delta CT values. (E) Cytokine secretion. (A) *TNF-α*, (B) *CCL5*, (C) *COX2*, (D) *TSG6*, and (E) IL-12p70. The Wilcoxon test was used for paired samples for gene expression, and the paired *t*-test was used for cytokine secretion. *p* Values < 0.05 were considered significant. The data are presented as the minimum to maximum values. obASCs, ASCs derived before weight loss; wlASCs, ASCs derived after weight loss.

ASCs are highly attractive for use in cellular therapies, particularly because of their immunomodulatory functions, in which cytokine secretion plays an important role [1]. However, Oñate et al. and Serena et al. previously explored the impact of weight loss on the gene expression of immunomodulatory factors in ASCs, and none have specifically investigated the same donor before and after weight loss [30, 69]. In this study, we examined the effects of weight loss on pro- and anti-inflammatory gene expression levels in ASC monocultures (Figure 3 and Figure S5). Our findings revealed significant downregulations in the expression of proinflammatory genes in wlASCs (*n* = 4) compared with obASCs (*n* = 4, *TNF-α*: *p*=0.0329, chemokine ligand 5 [*CCL5*]: *p*=0.0158, and cyclooxygenase-2 [*COX2*]: *p*=0.0076; Figure 3). Additionally, we observed a significant decrease in the expression of the anti-inflammatory gene tumor necrosis factor-inducible gene 6 (*TSG6*) in wlASCs (*p*=0.0378) compared with obASCs (Figure 3D).

#### 3.5.2. Decreased Secretion of the Proinflammatory Cytokine IL-12p70 by ASCs After Weight Loss

Obesity-induced inflammation alters cytokine secretion in adipose tissue [70], which changes the ASC cytokine profile to be more proinflammatory [35]. For example, obesity-induced inflammation elevates the levels of IL-12p70, a member of the IL-12 family, in both immune cells and adipocytes [71, 72]. However, cytokines of the IL-12 family have both proinflammatory and immunoregulatory functions [73, 74]. As reported by Verma et al. [75], IL-12p70 may also play a role in downregulating immune responses by inducing regulatory T cells. In this study, the overall secreted amounts of proinflammatory cytokines were low in both obASCs (*n* = 4) and wlASCs (*n* = 4) (Figure S6). However, compared with obASCs, wlASCs (*p*=0.0409) secreted significantly less IL-12p70 (Figure 3E). Additionally, nonsignificant decreases in the levels of all the studied anti-inflammatory cytokines, such as IL-4, IL-1RA, and IL-10, were observed after weight loss (Figure S6G–I). In line with our results, Silva et al. [35] reported that weight loss did not fully improve cytokine secretion by ASCs.

### 3.6. Downregulation of Both Pro- and Anti-Inflammatory Surface Markers in M1 Macrophages by obASCs and wlASCs in Cocultures

**Figure 4:**
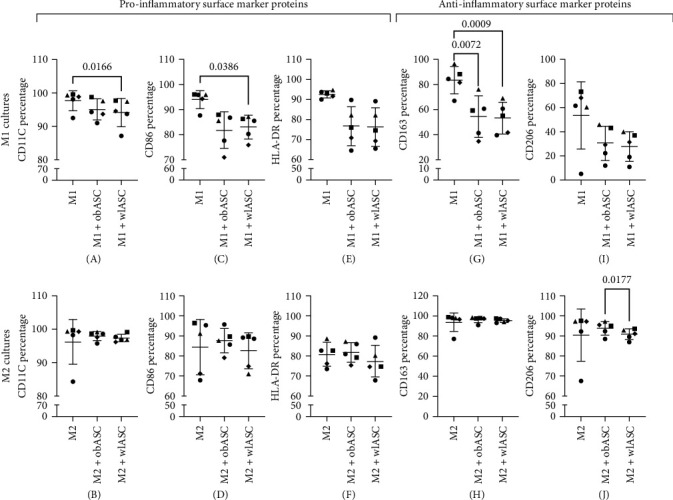
Percentages of CD markers in M1 and M2 cells in monoculture and coculture with obASCs and wlASCs; *n* = 5. (A) CD11C, (C) CD86, (E) HLA-DR, (G) CD163, and (I) CD206 in M1 mono-/cocultures. (B) CD11C, (D) CD86, (F) HLA-DR, (H) CD163, and (J) CD206 expression in M2 mono-/cocultures. For RM ANOVA, *p* values < 0.05 were considered significant. The data are presented as the means and SDs. M1, proinflammatory macrophages; M2, anti-inflammatory macrophages; obASCs, ASCs derived before weight loss; wlASCs, ASCs derived after weight loss. See also Figure S13.

Previous studies have indicated that the immunomodulatory capacity of ASCs is strongly linked to donor BMI. Specifically, conditioned media or exosomes from ASCs obtained from donors with normal weight have been shown to reduce inflammation and shift the macrophage phenotype from M1 to M2 both in vitro and in vivo [76–78]. However, ASCs derived from obese donors may promote M1 polarization [26] and exhibit a reduced or impaired ability to suppress M1 macrophages while activating M2 macrophages [26, 30]. In contrast to previous findings, our study revealed that ASCs did not direct M1 macrophages toward the M2 phenotype; that is, they did not function as anti-inflammatory cells. In our study, compared with those in M1 monocultures, downregulation of the proinflammatory cluster of differentiation (CD) markers CD11C and CD86 and the anti-inflammatory marker CD163 was observed in cocultures with both obASCs and wlASCs (Figure 4). Specifically, significant decreases in the percentages of CD11C (*p*=0.0166)- and CD86 (*p*=0.0386)-positive macrophages were observed in M1 cocultures with wlASCs compared with those in M1 monocultures (Figure 4A,C). Similarly, the percentage of CD163-positive macrophages was significantly lower in M1 cocultures with both obASCs (*p*=0.0072) and wlASCs (*p*=0.0009) than in M1 monocultures (Figure 4G).

### 3.7. Downregulation of the Anti-Inflammatory Marker CD206 in M2 Macrophages by wlASCs in Cocultures

Previous studies have indicated that ASCs can guide M0 macrophages toward the M2 phenotype [30]. Consequently, we anticipated that M2 macrophages would either maintain or enhance their anti-inflammatory phenotype when cocultured with ASCs, particularly wlASCs. However, contrary to our expectation, we observed that M2 macrophages repolarized toward a proinflammatory phenotype when they were cocultured with both types of ASCs. In our study, obASCs and wlASCs downregulated the anti-inflammatory marker CD206 in M2 macrophages. A significantly lower percentage of CD206-positive cells was observed in M2 cocultures with wlASCs (*p*=0.0177) than in M2 cocultures with obASCs (Figure 4J). Additionally, the MFI of CD206 was significantly lower in M2 cocultures with both obASCs (*p*=0.0083) and wlASCs (*p*=0.0061) than in M2 monocultures (Figure S13J). Furthermore, we also noted a significantly decreased MFI of HLA-DR in M2 cocultures with obASCs (*p*=0.0392) compared with M2 monocultures (Figure S13F).

### 3.8. Pro- and Anti-Inflammatory Gene Expression and Cytokine Secretion in M1 Macrophage Cocultures

#### 3.8.1. Inconsistent Regulation of Pro- and Anti-Inflammatory Gene Expression in M1 Macrophages by obASCs and wlASCs in Cocultures

**Figure 5:**
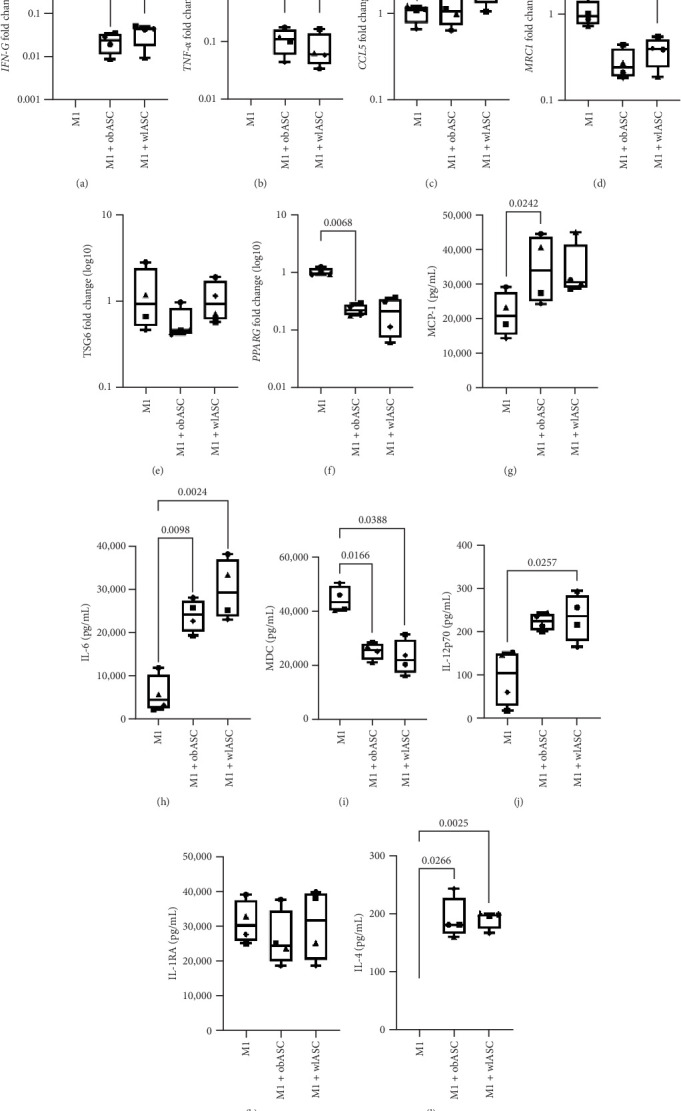
Comparison of gene expression and cytokine secretion between mono- and cocultures of M1 macrophages with obASCs and wlASCs; *n* = 4. (A)–(F) Gene expression in M1 mono-/cocultures. (G)–(L) Cytokine secretion in M1 mono-/cocultures. (A) *IFNɣ*, (B) *TNF-α*, (C) *CCL5*, (D) *MRC1*, (E) *TSG6*, (F) *PPAR-ɣ*, (G) MCP-1, (H) IL-6, (I) MDC, (J) IL-12p70, (K) IL-1RA, and (L) IL-4. For RM ANOVA, *p* values < 0.05 were considered significant. The data are presented as the minimum to maximum values. M1, proinflammatory macrophages; obASCs, ASCs derived before weight loss; wlASCs, ASCs derived after weight loss. See also Figure S14.

In contrast to our surface marker results, we observed significant decreases in the expression levels of proinflammatory genes, specifically *IFNɣ* and *TNF-α*, in macrophage cocultures with both obASCs and wlASCs (Figure 5). Compared with their corresponding macrophage monocultures, the M1 cocultures with obASCs and wlASCs (obASCs; *p*=0.0151 and wlASCs; *p*=0.0343) presented significantly lower *IFNɣ* expression (Figure 5A). Similarly, the expression of *TNF-α* was significantly lower in M1 cocultures (obASCs; *p*=0.0058 and wlASCs; *p*=0.0176) than in their respective macrophage monocultures (Figure 5B).

Interestingly, *CCL5*, which is associated with macrophage recruitment and M1 polarization during obesity-induced inflammation [79–81], exhibited a different pattern. In our study, the expression of *CCL5* was significantly increased in M1 cocultures with both obASCs and wlASCs (obASCs; *p*=0.0088 and wlASCs; *p*=0.0025) (Figure 5C). This result contrasts with those of previous studies suggesting that ASCs possess anti-inflammatory abilities that downregulate *CCL5* expression [82]. However, a recent study reported that ASC-mediated *CCL5* plays a crucial role in T-cell accumulation within adipose tissue, antagonizing obesity-induced inflammation [83].

In our study, we observed a modest downregulation of anti-inflammatory gene expression in M1 cocultures with both obASCs and wlASCs (Figure 5 and Figure S14). Compared with M1 monocultures, M1 macrophages cocultured with wlASCs presented significantly lower mannose receptor C-type 1 (*MRC1*) expression (*p*=0.0382) (Figure 5D). In M1 cocultures with obASCs, peroxisome proliferator-activated receptor-*γ* (*PPARɣ*) expression was significantly lower (*p*=0.0068) than that in M1 monocultures (Figure 5F). Similarly, *KLF-4* expression in M1 cocultures with obASCs was significantly lower (*p*=0.0061) than that in M1 monocultures (Figure S14B). Furthermore, compared with M1 monocultures, M1 cocultures with obASCs presented significantly decreased human leukocyte antigen G (*HLA-G*) expression (Figure S14F).

#### 3.8.2. Increased Secretion of Proinflammatory Cytokines by ASCs Before and After Weight Loss in M1 Cocultures

In M1 cocultures with both obASCs and wlASCs (Figure 5), there were notable increases in the secretion of the proinflammatory cytokines MCP-1, IL-6, IL-12p70, and IL-1*β*. IL-12p70 and IL-1*β* secretion levels were significantly greater, particularly in wlASCs, than in M1 monocultures. We observed significantly increased secretion of MCP-1 in M1 cocultures with obASCs (*p*=0.0242) compared with M1 monocultures (Figure 5G). These results align with previous findings by Silva et al. [35], where MCP-1 secretion was notably greater in M1 cocultures with obASCs than in those with ASCs from normal-weight donors. Similarly, the secretion levels of IL-12p70 (*p*=0.0257, Figure 5J) and IL-1*β* (*p*=0.0352, Figure S14H) were significantly greater in M1 cocultures with wlASCs than in M1 monocultures. In contrast, a significant decrease in the secretion of the proinflammatory chemokine MDC was observed in M1 cocultures with ASCs (obASCs; *p*=0.0166 and wlASCs; *p*=0.0388) compared with M1 monocultures (Figure 5I).

The secretion levels of IL-6 and IL-4 were significantly greater in both obASC (IL-6; *p*=0.0098 and IL-4; *p*=0.0266) and wlASC (IL-6; *p*=0.0024 and IL-4; *p*=0.0025) cocultures than in M1 monocultures (Figure 5H, L, respectively). Previous studies have highlighted the pleiotropic role of IL-6 secretion by MSCs in mediating immunomodulatory responses, particularly during inflammation [16, 84, 85]. Interestingly, an in vivo mouse study reported that IL-6 secretion from adipocytes derived from donors with obesity could stimulate adipose tissue macrophages, leading to IL-4 signaling and contributing to an anti-inflammatory environment [86]. In our study, we observed simultaneous increases in both IL-6 and IL-4 secretion. Therefore, we speculate that the increased secretion of IL-6 plays a role in the concurrent increase in IL-4 secretion in the cocultures.

### 3.9. Pro- and Anti-Inflammatory Gene Expression and Cytokine Secretion in M2 Macrophage Cocultures

#### 3.9.1. Inconsistent Regulation of Pro- and Anti-Inflammatory Gene Expression and Cytokine Secretion in M2 Macrophages by obASCs and wlASCs in Cocultures

**Figure 6:**
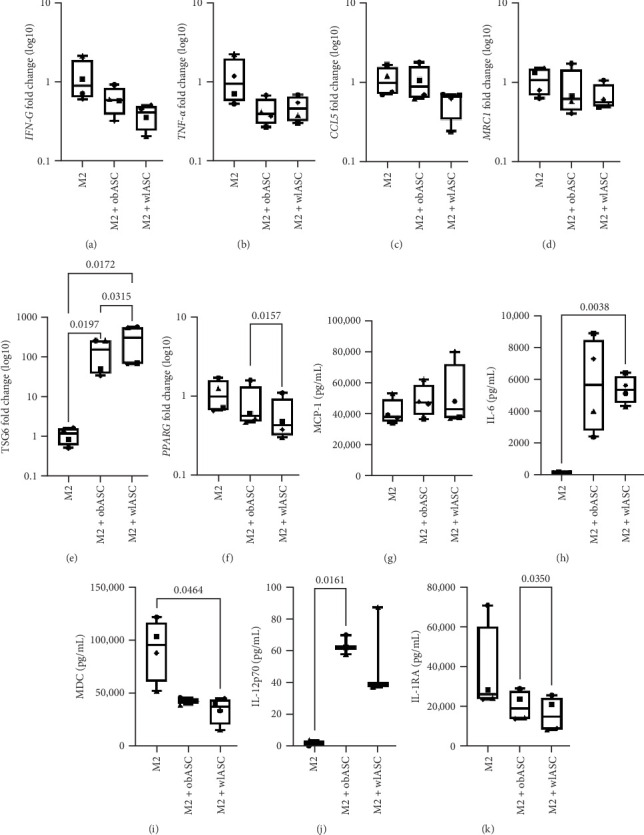
Comparison of gene expression and cytokine secretion between mono- and cocultures of M2 macrophages with obASCs and wlASCs; *n* = 4. (A)–(F) Gene expression in M2 mono-/cocultures. (G)–(K) Cytokine secretion in M2 mono-/cocultures. (A) *IFNɣ*, (B) *TNF-α*, (C) *CCL5*, (D) *MRC1*, (E) *TSG6*, (F) *PPAR-ɣ*, (G) MCP-1, (H) IL-6, (I) MDC, (J) IL-12p70, and (K) IL-1RA. For RM ANOVA, *p* values < 0.05 were considered significant. The data are presented as the minimum to maximum values. M2, anti-inflammatory macrophages; obASCs, ASCs derived before weight loss; wlASCs, ASCs derived after weight loss. See also Figure S15.

With respect to the M2 cocultures, we did not observe any significant differences in proinflammatory gene expression (Figure 6A–C). Like in M1 cocultures, the expression of anti-inflammatory genes was slightly downregulated in M2 cocultures with both obASCs and wlASCs (Figure 6 and Figure S15). Notably, *TSG6*, a protein secreted by ASCs, plays a crucial role in regulating macrophage function and reprograms them toward an anti-inflammatory phenotype when in contact with ASCs [87–91]. In line with these findings, we observed that *TSG6* expression was significantly greater in M2 cocultures with ASCs (obASCs; *p*=0.0197 and wlASCs; *p*=0.0172) than in M2 monocultures. Additionally, *TSG6* expression was slightly but significantly greater in M2 cocultures with wlASCs (*p*=0.0315) than in M2 cocultures with obASCs (Figure 6E). In an inflammatory environment, MSC expression of *TSG6* increases, especially in contact with macrophages, leading to attenuated inflammation and enhanced tissue repair [87, 92–96].

*PPARɣ*, a critical player in macrophage polarization toward the M2 phenotype, serves as a key regulator of the inflammatory potential of tissue macrophages [97]. Moreover, in AT, obesity-induced inflammation downregulates *PPARɣ* expression in M2 macrophages, exacerbating the inflammatory milieu [98]. Restoring *PPARɣ* expression is essential for reinstating the anti-inflammatory functions of both macrophages and adipocytes, which become compromised during obesity-induced inflammation [99]. Surprisingly, contrary to previous findings, our study revealed that weight loss did not lead to the restoration of *PPARɣ* expression. Notably, in M2 cocultures with wlASCs, *PPARɣ* expression was significantly lower (*p*=0.0157) than that in M2 cocultures with obASCs (Figure 6F). Consistent with *PPARɣ* expression, M2 cocultures with wlASCs presented markedly reduced levels of the anti-inflammatory protein *HLA-G* (Figure S15F), the secretion of which has been reported to enhance anti-inflammatory and immunosuppressive responses in an inflammatory environment [100].

#### 3.9.2. Similar Regulation of Cytokine Secretion by ASCs Before and After Weight Loss in M2 Cocultures

Like M1 cocultures, M2 cocultures with wlASCs (*p*=0.0464) exhibited significantly reduced secretion of the proinflammatory chemokine MDC compared with their respective monocultures (Figure 6I). Furthermore, increased secretion levels of the proinflammatory cytokines IL-6 and IL-12p70 were observed in M2 cocultures with both obASCs and wlASCs (Figure 6). The secretion of IL-6 was significantly greater in M2 cocultures with wlASCs (wlASCs; *p*=0.0038) than in M2 monocultures (Figure 6H). Similarly, the secretion of IL-12p70 was significantly greater in M2 cocultures with obASCs (obASCs; *p*=0.0161) than in M2 monocultures (Figure 6J). In contrast to M1 cocultures, significantly increased secretion of anti-inflammatory IL-1RA was observed in M2 cocultures with obASCs (*p*=0.0350) compared with M2 cocultures with wlASCs (Figure 6K). These findings diverge from those of previous studies where ASCs derived from nonobese donors (BMI < 30 kg/m^2^) not only polarized M1 macrophages toward M2 macrophages but also enhanced the anti-inflammatory properties of M2 macrophages when cultured in ASC-conditioned medium [77]. Notably, IL-4 quantification for M2 macrophages was not included in the results, as IL-4 was utilized for their polarization and activation.

## 4. Discussion

Human ASCs are appealing candidates for cell-based therapies [1–4]. However, certain properties of ASC donors, such as obesity, have been found to negatively impact ASC characteristics and their immunomodulatory functions [16, 26, 63], thereby limiting their clinical potential. However, weight loss has been shown to restore individuals' metabolic health [45, 47, 48] and improve ASC properties [33–36].

Consistent with previous studies, in our study, we observed that weight loss led to improved plasma inflammatory marker levels and reduced adipose tissue inflammation. Additionally, we detected changes in ASC characteristics or functions, such as mitochondrial respiration, the CD marker profile, proliferation, and adipogenesis, in accordance with previous findings [52, 58, 60, 63].

In line with Onate et al. and Serena et al. [30, 69] in wlASC monocultures, we observed significant downregulations in the expression of all studied proinflammatory genes. Additionally, the secretion of the proinflammatory cytokine IL-12p70 was reduced in ASCs following weight loss. However, contrary to earlier findings, the expression of the anti-inflammatory gene *TSG6* was also significantly downregulated in our study. Notably, La Rusa et al. reported that *TSG6*, which is secreted by MSCs, plays preventive and immunomodulative roles in various inflammatory diseases [90].

In contrast to prior studies [76–78], neither obASCs nor wlASCs directed inflammatory M1 macrophages toward an anti-inflammatory M2 phenotype. Interestingly, earlier research suggested that ASCs guide primary human adipose tissue-derived M0 macrophages toward the M2 phenotype [30]. Based on these findings, we hypothesized that M2 macrophages would either maintain or enhance their anti-inflammatory phenotype when cocultured, especially with wlASCs. In contrast, our observations revealed that M2 macrophages were repolarized toward a proinflammatory phenotype in coculture with both obASCs and wlASCs.

While numerous of our macrophage coculture results suggest increased proinflammatory and decreased immunosuppressive properties with both obASCs and wlASCs, there are notable exceptions that may reflect the transitional state of these cells. For example, in M1 cocultures with wlASCs, we observed significant downregulation of the proinflammatory markers CD11C and CD86. Similarly, the secretion of the proinflammatory chemokine MDC was significantly reduced in cocultures of M1 macrophages with both obASCs and wlASCs, and in M2 macrophage cocultures with wlASCs, whereas the secretion of the anti-inflammatory cytokine IL-4 was significantly increased in M1 cocultures with both obASCs and wlASCs. These results partially align with a study by Lopes-Alves et al., where ASCs obtained from previously obese donors were able to induce the M2 phenotype, although their secretory profile was a mix of M1 and M2 [31]. Despite significant weight loss, our ASC donors remained overweight or obese, which could account for the conflicting results compared with those obtained with ASCs derived from normal-weight donors. Additionally, in our study, ASCs were obtained from the same donors before and after bariatric surgery-related weight loss, which is a unique study setting that has not been published previously.

The divergences in experimental designs may also play a role in the conflicting results obtained in different studies. For example, in contrast to previous work by Serena et al. [30], where human primary adipose tissue-derived M0 macrophages were cultured in an ASC-conditioned medium, our study focused on coculturing obASCs and wlASCs with prepolarized and preactivated human M1 and M2 macrophages derived from PBMCs. This approach aims to mimic the inflammatory environment of adipose tissue, where macrophages exist in varying states of inflammation [22, 23]. Wang et al. [61] had similar study settings in terms of macrophage polarization as those used in our study. However, they studied the paracrine effects of human ASCs from normal-weight donors versus obese donors on mouse macrophages and reported increased expression of proinflammatory M1 markers when cocultured with obASCs but no changes in M2-related markers [61]. Similarly, Harrison et al. [26] reported reduced immunomodulatory effects of human obASCs on murine macrophages and microglia with upregulated expression of proinflammatory genes compared with those of normal-weight donor-derived ASCs. In our study, coculture time points were chosen based on previous studies on the immunomodulatory properties of MSCs to analyze MSC-macrophage interactions [26, 42, 61]. However, variations in coculture duration may influence the results and should be considered when interpreting the findings.

Furthermore, in our study, after isolation from adipose tissue, ASCs were cultured in a medium containing human serum rather than FBS, which has been utilized in other studies [26, 30]. However, in our study, FBS was also utilized in cocultures with macrophages. In our preliminary experiments, the macrophages were not viable in a medium containing human serum; therefore, we cocultured the ASCs and macrophages in a medium containing FBS. In addition, the sample collection time after weight loss might have affected the results, as there might be changes in the weight and metabolic functions of the donors over the weight loss period. However, our schedule of obtaining samples after bariatric surgery was quite similar to that used in a previous study; for example, 2–4 years resulted in similar cytokine profiles [35]. In line with the findings of Silva et al., the cytokine profile of ASCs in our study did not fully recover after weight loss.

In this study, our aim was to compare the effects of weight loss on the characteristics and functions of ASCs obtained from the same donors before and after weight loss, which led to a small sample size. The small sample size of the groups is a study limitation; larger study groups may be needed to confirm our results. In addition, weight loss was facilitated by bariatric surgery, which might have affected the results. Bariatric surgery-related weight loss impacts adipose tissue physiology [57, 101]. However, the current literature does not provide comparisons between weight loss methods, that is, weight loss driven by lifestyle changes such as diet and exercise or the use of drugs such as semaglutide and how these methods affect ASCs. Furthermore, most of our ASC donors were females, presumably of Caucasian origin and within the same age range of 39–53 years; hence, more samples representing both sexes, different ethnicities, and wider age ranges might be needed to reveal their concomitant effects on weight loss. Importantly, despite experiencing considerable weight loss, all the donors were still either overweight or obese. Hence, a study group representing individuals with constant normal weight and a group that has reached normal weight after weight loss should be included in future studies to draw more definite conclusions about the effects of weight loss on ASC characteristics and functions.

The selection of donors for ASC-based allogeneic therapies should be guided by a comprehensive assessment of donor health, as various biological and lifestyle factors influence ASC function. Previous studies have demonstrated that in addition to donor obesity [16, 25–27] and weight loss [31, 34, 35], donor age [102–104], gender [105, 106], disease conditions [30, 107], and lifestyle factors [108] can impact the regenerative capacity and therapeutic potential of ASCs. Among these factors, BMI and weight loss history are particularly relevant, as they are associated with functional changes in ASCs that may influence their immunomodulatory properties and regenerative efficacy. However, BMI and weight loss history should not be considered in isolation but rather as part of a broader donor profiling strategy that integrates multiple biological and metabolic parameters. For instance, assessing inflammatory markers, metabolic health, or ASC functional assays could provide a more precise evaluation of donor suitability for ASC-based therapies.

## 5. Conclusions

In our study, in individuals with obesity, inflammation within adipose tissue strongly affected ASCs, influencing their gene expression and cytokine secretion and impairing their anti-inflammatory functions.

Bariatric surgery led to a significant reduction in BMI and improved individuals' metabolic health. This approach alters the composition of adipose tissue and impacts adipose tissue-resident ASCs by increasing their overall cellular respiration and proliferation capacity while decreasing their ability to differentiate into adipocytes.

Our study underscores the importance of examining multiple pro- and anti-inflammatory factors when investigating the impact of donor weight on the immunomodulatory functions of ASCs. The impact of weight loss on the immunomodulatory functions of ASCs varied according to the markers analyzed. Following weight loss, ASCs exhibited increased proinflammatory properties when cocultured with macrophages, characterized by the downregulation of anti-inflammatory factors, along with the upregulation of several proinflammatory factors, compared with those in monocultures. Conversely, wlASCs also demonstrated improved immunosuppressive functions in coculture with macrophages, as indicated by the upregulation of *TSG6* gene expression and IL-4 secretion. We could assume that, as the ASC donors are still overweight, ASCs have not resumed their normal functions and have mixed immunomodulatory behavior. Thus, despite extensive weight loss enhancing ASC metabolism, it remains insufficient to fully restore the immunosuppressive functions of ASCs.

To enhance clinical decision-making, further in vivo studies are needed to determine how donor characteristics, including BMI and weight loss history, translate into therapeutic outcomes. Establishing standardized criteria for ASC donor selection based on functional rather than solely anthropometric parameters will be essential for optimizing the safety and efficacy of ASC-based interventions.

## Data Availability

The data that support the findings of this study are available in the supporting information of this article.

## Nomenclature

ASCs: Adipose stromal/stem cells
MSCs: Mesenchymal stem cells
BMI: Body mass index
obASCs: ASCs obtained from donors with obesity
wlASCs: ASCs obtained from the same donors after weight loss
M1: Proinflammatory macrophages
M2: Anti-inflammatory macrophages
CRP: C-reactive protein
CLSs: Crown-like structures
OCR: Oxygen consumption rate
PBMCs: Peripheral blood mononuclear cells
IFN*ɣ*: Interferon gamma
LPS: Lipopolysaccharides
GM-CSF: Granulocyte-macrophage colony-stimulating factor
M-CSF: Macrophage colony-stimulating factor
FBS: Fetal bovine serum
ELISA: enzyme-linked immunosorbent assay
ISCT: International Society for Cell & Gene Therapy
IFATS: International Federation for Adipose Therapeutics and Science
MFI: Median fluorescence intensity
qRT–PCR: Quantitative real-time reverse transcription polymerase chain reaction
mRNA: Messenger RNA
CD: Cluster of differentiation
HLA-DR: Human leukocyte antigen–DR isotype
TNF-*α*: Tumor necrosis factor alpha
CCL5: Chemokine ligand 5
COX2: Cyclooxygenase-2
TSG6: Tumor necrosis factor-inducible gene 6
STAT6: Signal transducer and activator of transcription 6
MRC1: Mannose receptor C-type 1
IDO-1: Indoleamine 2,3-dioxygenase 1
PPARɣ: Peroxisome proliferator-activated receptor-*γ*
KLF4: Kruppel-like factor 4
HLA-G: Human leukocyte antigen G
IL-12p70: Interleukin 12p70
IL-1RA: Interleukin 1 receptor antagonist
IL-4: Interleukin 4
IL-10: Interleukin 10
MCP-1: Monocyte chemoattractant protein-1
IL-6: Interleukin 6
IL-1*β*: Interleukin 1 beta
MDC: Macrophage-derived chemokine
TARC: Thymus- and activation-regulated chemokine
MIP-1*α*: Macrophage inflammatory protein 1 alpha.

## Supporting Information

Supporting Information Table S1. Characteristics of the ASCs donors in the study. Table S2. Antibodies for the surface marker expression analysis. Proliferation and Multipotent Differentiation Capacity. Osteogenic Differentiation. Chondrogenic Differentiation. Adipogenic Differentiation. Proliferation Analysis of ASCs. Table S3. Composition of polarization and activation media for macrophage polarization assay. Figure S1. Graphical illustration of macrophage polarization and activation assay's timeline. Table S4. TaqMan assay probes and likely source of genes used for qRT PCR. Table S5. V-plex assay kits for cytokine analysis. Table S6. Statistical analysis of donor variables before and after the weight loss. Figure S2. The effect of weight loss on adipocyte area and crown-like structures (CLS). Figure S3. Comparison of different surface marker expressions between obASCs and wlASCs. Figure S4. Comparison of different surface marker expressions between obASCs and wlASCs. Figure S5. Comparison of gene expression between obASCs and wlASCs. Figure S6. Comparison of cytokine secretion between obASCs and wlASCs. Additional Result: Multipotent Differentiation Capacity of obASCs and wlASCs. Figure S7. Multipotent differentiation capacity of ASCs before and after the weight loss. Additional Result: Increased Proliferation after Weight loss. Figure S8. Proliferation capacity of ASCs before and after the weight loss. Additional Result: Cytochemical Staining. Additional Result: Phenotypic Characterization of Macrophage. Figure S9. Morphology of different macrophages after 6 days culture in 10% FBS medium. Figure S10. Representative gating images of M1 and M2 type macrophages from one experiment. Figure S11. Surface markers of M1 and M2 type macrophages. Figure S12. Representative gating images of M1 and M2 type macrophages with ASC cocultures from one donor. Figure S13. MFI of CD markers in M1 and M2 cells in monoculture and coculture with obASCs and wlASCs. Figure S14. Comparison of gene expression and cytokine secretion between mono- and cocultures of M1 with obASCs and wlASCs. Figure S15. Comparison of gene expression and cytokine secretion between mono- and cocultures of M2 with obASCs and wlASCs. Supporting Information References.
