# Supplementary material for: Effects of Bariatric Surgery-Related Weight Loss on the Characteristics, Metabolism, and Immunomodulation of Adipose Stromal/Stem Cells in a Follow-Up Study
Source: Stem Cells Int. 2025 May 13;2025:1212255. doi: 10.1155/sci/1212255 (PMC12092157; doi:10.1155/sci/1212255)
Supplement: Supporting Information — Table S1. Characteristics of the ASCs donors in the study. Table S2. Antibodies for the surface marker expression analysis. Proliferation and Multipotent Differentiation Capacity. Osteogenic Differentiation. Chondrogenic Differentiation. Adipogenic Differentiation. Proliferation Analysis of ASCs. Table S3. Composition of polarization and activation media for macrophage polarization assay. Figure S1. Graphical illustration of macrophage polarization and activation assay's timeline. Table S4. TaqMan assay probes and likely source of genes used for qRT PCR. Table S5. V-plex assay kits for cytokine analysis. Table S6. Statistical analysis of donor variables before and after the weight loss. Figure S2. The effect of weight loss on adipocyte area and crown-like structures (CLS). Figure S3. Comparison of different surface marker expressions between obASCs and wlASCs. Figure S4. Comparison of different surface marker expressions between obASCs and wlASCs. Figure S5. Comparison of gene expression between obASCs and wlASCs. Figure S6. Comparison of cytokine secretion between obASCs and wlASCs. Additional Result: Multipotent Differentiation Capacity of obASCs and wlASCs. Figure S7. Multipotent differentiation capacity of ASCs before and after the weight loss. Additional Result: Increased Proliferation after Weight loss. Figure S8. Proliferation capacity of ASCs before and after the weight loss. Additional Result: Cytochemical Staining. Additional Result: Phenotypic Characterization of Macrophage. Figure S9. Morphology of different macrophages after 6 days culture in 10% FBS medium. Figure S10. Representative gating images of M1 and M2 type macrophages from one experiment. Figure S11. Surface markers of M1 and M2 type macrophages. Figure S12. Representative gating images of M1 and M2 type macrophages with ASC cocultures from one donor. Figure S13. MFI of CD markers in M1 and M2 cells in monoculture and coculture with obASCs and wlASCs. Figure S14. Comparison of gene expression [file 1212255.f1.zip › Additional file 1.docx]

**Additional Table 1. Characteristics of the ASCs donors in the study**

| **Donor cell line code** | **Age** | **Sex** | **∆BMI** | **BMI before**  **(kg/m2)** | **BMI after**  **(kg/m2)** | **Time between samples** |
| --- | --- | --- | --- | --- | --- | --- |
| D1 | 45 | Female | 28.61 | 55.02 | 26.41 | 38 months |
| D2 | 52 | Female | 11.29 | 42.75 | 31.46 | 27 months |
| D3 | 52 | Female | 11.01 | 40.06 | 29.05 | 26 months |
| D4 | 39 | Female | 11.2 | 39.91 | 28.71 | 26 months |
| D5 | 39 | Female | 15.62 | 42.39 | 26.77 | 34 months |
| D6 * | 53 | Male | 14.71 | 39.89 | 25.18 | 35 months |

Abbreviation. BMI: body mass index. *Surface marker expression data not included

**Additional Table 2. Antibodies for the surface marker expression analysis**

| **ANTIBODIES** | **SOURCE** | **IDENTIFIER** |
| --- | --- | --- |
| BV421 Mouse Anti-Human CD13 | BD Biosciences | 562596 |
| APC Mouse Anti-Human CD14 | BD Biosciences | 561708 |
| APC Mouse Anti-Human CD19 | BD Biosciences | 561742 |
| Anti human CD29 MEM-101A IgG1 FITC | Immunotools | 21270293 |
| BV421 Mouse Anti-Human CD31 | BD Biosciences | 564089 |
| APC- conjugated monoclonal antibody to human CD34 | Immunotools | 21270346 |
| APC Mouse Anti-Human CD36, Clone CB38 | BD Pharmingen | 561822 |
| FITC Mouse Anti-Human CD44 | BD Biosciences | 560977 |
| CD45RO-APC | BD Biosciences | 340438 |
| Human ICAM-1/CD54 Fluorescein-conjugated Antibody | R&D Systems | BBA20 |
| FITC Mouse Anti-Human CD73 | BD Biosciences | 561254 |
| APC Mouse Anti-Human CD90 | BD Pharmingen | 561971 |
| FITC Mouse anti-Human CD105 | BD Biosciences | 561443 |
| BV421 Mouse Anti-Human CD146 | BD Biosciences | 566226 |
| BV421 Mouse Anti-Human CD235a | BD Biosciences | 562938 |
| BV421 Mouse anti-human HLA-DR | BD Biosciences | 562804 |
| PE-CF594 Mouse Anti-Human CD163 | BD | 562670 |
| APC Mouse Anti-Human CD206 | BD | 550889 |
| PE-CF594 Mouse Anti-Human CD86 | BD | 562390 |
| PE-Cy™7 Mouse Anti-Human CD11c | BD Pharmingen | 561356 |

**Proliferation and Multipotent Differentiation Capacity**

To verify the proliferation and multipotential differentiation capacity of ASCs, obASCs (n=5) and wlASCs (n=5) were differentiated into adipogenic, osteogenic, and chondrogenic lineages [1].

**Osteogenic Differentiation**

Osteogenic differentiation (OD) result of studied donor adipose stromal/stem cells at passage 5 or 6. Differentiation medium composition and culture conditions are described previously [2]. Cells were cultured in a 24 well plate for about 3 weeks in OD medium supplemented with bioactive glass (S53P4 BaG granules (500-1000µm), which was changed twice a week. Differentiated ASCs were stained with Alizarin Red S (Sigma) to study the cellular potential of producing mineralized calcium deposits. Samples were photographed with Canon Digital IXUS 100IS camera.

**Chondrogenic Differentiation**

Chondrogenic differentiation (CD) result of studied donor adipose stromal/stem cells at passage 5 or 6. Medium composition and culture conditions are briefly described as; 8 × 10^4^ cells were seeded on a 24-well culture plate in a 10-μl volume and were allowed to adhere for 3 hrs before the addition of chondrogenic induction medium (DMEM F/12 + 1% Glutamax, 100 U/ml penicillin and 100µg/ml streptomycin, 1x ITS+1; Sigma, 50µg/ml Ascorbic Acid-2-phosphate; Sigma, 55µg/ml Na-pyruvate; Lonza, 23µg/ml L-proline; Sigma and 2µg/ml TGF-β1; Sigma). Medium was changed twice a week. After 14 days of chondrogenic induction, differentiation was confirmed by using the Alcian blue (Sigma) staining method. For that, ASCs pellets were rinsed with DPBS and fixed with 4% Paraformaldehyde (PFA). Subsequently, cells were rinsed twice with deionized water and stored in 70% ethanol. Pellets were dehydrated, embedded in paraffin, and sectioned at 4µm thickness. The sections were rehydrated and stained with Alcian blue (pH 1.0) to detect sulfated glycosaminoglycans (GAGs) by using Nuclear Fast Red - aluminum sulfate solution (Sigma) as a counterstain to study the cellular potential of producing GAGs (scale bar; 100 µm). Samples were photographed with microscope scanner (Hamamatsu S60).

**Adipogenic Differentiation**

Adipogenic differentiation (AD) result of studied donor adipose stromal/stem cells at passage 5 or 6. Medium composition and culture conditions are briefly described as; 7.5 × 10^4^ cells/well were seeded on a 24 well culture plate for 24 hrs. Next day growth medium was replaced with differentiation medium Dulbecco's modified Eagle's medium/Nutrient Mixture F-12 (DMEM/F12; Gibco, 3% HS; Serana, 100 U/ml penicillin and 100µg/ml streptomycin; Lonza, 100 nM Insulin; Gibco, 1 *μ*M Dexamethasone; Sigma, 0.5 mM 3-isobutyl-1-methylxanthine; Sigma, 1 *μ*M Rosiglitazone; MERCK Millipore, 33 *μ*M Biotin; Sigma and 17 *μ*M Pantothenate; MERCK). Induction was performed for 7 days. After that differentiation medium was changed to maintenance medium (DMEM/F12, 3% HS, 100 nM Insulin, 1 *μ*M Dexamethasone, 33 *μ*M Biotin and 17 *μ*M Pantothenate). Duration for maintenance medium was for 7-14 days, medium was changed in every 4-5 days. During the differentiation process, cells were cultured for about 3 weeks. Differentiated ASCs were stained with Oil Red O to study the cellular potential of lipid accumulation (scale bar; 100 µm). ASCs cultured in control medium (DMEM F/12 + 1% Glutamax with 5% HS, 100 U/ml penicillin and 100µg/ml streptomycin) were used as negative control. Samples were photographed with a light microscope (NikonEclipse TE2000-S).

**Proliferation Analysis of ASCs**

Proliferation rate of obASCs (n=5) and wlASCs (n=5) was analyzed with a Cell Counting Kit-8 (CCK-8) assay (Dojindo Laboratories) according to manufacturer’s instructions. Briefly, cells were seeded at the density of 500 cells/cm^2^ in a 48 well plate (Nunc™, Thermo Scientific™) and proliferation was measured at day 7, 9 and 11 in basic medium. Culture medium was replaced with DPBS and CCK-8 reagent at 10:1 ratio. Absorbance was measured at 450nm with a microplate reader (VICTOR Nivo) after a 3hrs incubation at 37°C, 5% CO_2_.

**Additional Table 3. Composition of polarization and activation media for macrophage polarization assay**

| **Cell type** | **Polarization media** | **Activation media** |
| --- | --- | --- |
| M1 | 50ng/ml recombinant human granulocyte/macrophage colony stimulating factor (rhu GM-CSF), 10% FBS in RPMI-1640+1% P/S | 50ng/ml IFNɣ, 10 ng/ml lipopolysaccharide from E. coli (LPS) and 50 ng/ml GM-CSF, 10 % FBS in RPMI+1% P/S |
| M2 | 50ng/ml recombinant human macrophage colony stimulating factor (rhu M-CSF), 10% FBS in RPMI-1640+1% P/S | 20ng/ml IL-4 (Gibco), 50ng/ml rhu M-CSF, 10% FBS in RPMI-1640+1% P/S |

Abbreviations. M1: pro-inflammatory macrophage, M2: Anti-inflammatory macrophage, rhuGM-CSF: recombinant human granulocyte/macrophage colony stimulating factor, IFNɣ: Interferon gamma, LPS: lipopolysaccharide, FBS: Fetal bovine serum, P/S: Penicillin/Streptomycin, rhu M-CSF: recombinant human macrophage colony stimulating factor, IL-4: interleukin-4.

**
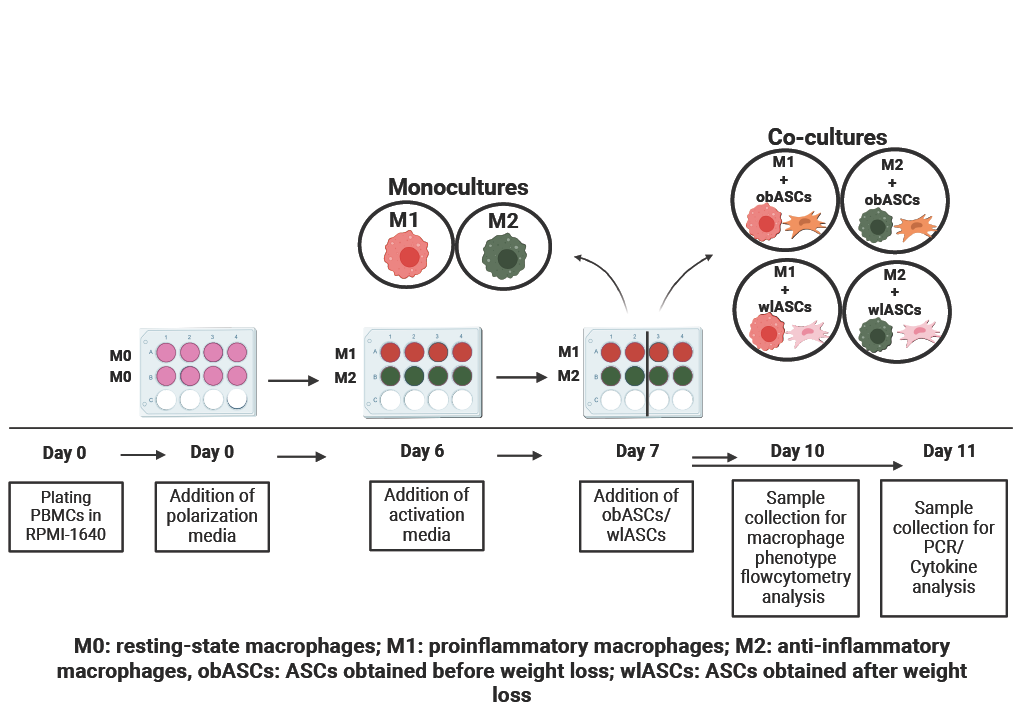
**

**Additional Figure 1. Graphical illustration of macrophage polarization and activation assay’s timeline.** Created in BioRender. Adnan, A. (2025) https://BioRender.com/wt05eef

**Additional Table 4. TaqMan assay probes and likely source of genes used for qRT PCR.**

| **Gene symbol** | **Gene name, alias** | **TaqMan Assay ID** |
| --- | --- | --- |
| *18s rRNA* | Eukaryotic 18S rRNA | Hs99999901_s1 |
| *GAPDH* | glyceraldehyde-3-phosphate dehydrogenase | Hs02786624_g1 |
| *COX2* | prostaglandin-endoperoxide synthase 2 | Hs00153133_m1 |
| *CCL5* | C-C motif chemokine ligand 5 | Hs00982282_m1 |
| *TNF-α* | tumor necrosis factor | Hs00174128_m1 |
| *CD163* | CD163 molecule | Hs00174705_m1 |
| *PPARG* | peroxisome proliferator activated receptor gamma | Hs01115513_m1 |
| *TSG6* | TNF alpha induced protein 6 | Hs00200180_m1 |
| *KLF4* | Kruppel like factor 4 | Hs00358836_m1 |
| *HLA-G* | major histocompatibility complex, class I, G | Hs00365950_g1 |
| *IDO1* | indoleamine 2,3-dioxygenase 1 | Hs00984148_m1 |
| *STAT6* | signal transducer and activator of transcription 6 | Hs00598625_m1 |
| *MRC1* | mannose receptor, C type 1 | Hs07288635_g1 |
| *IFNG* | interferon gamma | Hs00989291_m1 |

Abbreviation. qRT PCR: Quantitative real-time reverse-transcription PCR

**Additional Table 5. V-plex assay kits for cytokine analysis**

| **Utilized kits (dilution)** | **Cytokines** | **Detection range (pg/ml)** |
| --- | --- | --- |
| Cytokine Panel 2 Human kit (250-fold) | Interleukin- 1 receptor antagonist protein  (IL-1RA) | 1.12 – 650 |
| Chemokine Panel 1 Human kit (100-fold) | Thymus- and activation-regulated chemokine (TARC) | 0.22 - 1,120 |
|  | Macrophage inflammatory protein-1 alpha  (MIP-1α) | 3.02 - 743 |
|  | Macrophage-derived chemokine (MDC) | 1.22 - 3,700 |
|  | Monocyte chemoattractant protein-1 (MCP-1) | 0.09 - 375 |
| Proinflammatory Panel 1 Human kit (10-fold) | Interleukin-1 beta (IL-1β) | 0.05 - 375 |
|  | Interleukin-4 (IL-4) | 0.02 - 158 |
|  | Interleukin-6 (IL-6) | 0.06 - 488 |
|  | Interleukin-10 (IL-10) | 0.04 - 233 |
|  | Interleukin-12p70 (IL-12p70) | 0.11 - 315 |
|  | Tumor necrosis factor alpha (TNF-α) | 0.04 - 248 |

Abbreviation. pg/ml: picograms per milliliter.

**Additional Table 6. Statistical analysis of donor variables before and after the weight loss.**

| **Donor variable** | **Before weight loss (n=6)** | | | **After weight loss (n=6)** | | | ***p value*** |
| --- | --- | --- | --- | --- | --- | --- | --- |
|  | **Median** | **Minimum** | **Maximum** | **Median** | **Minimum** | **Maximum** |  |
| **Age** | 48.50 | 39.00 | 53.00 | 51.00 | 41.00 | 56.00 | 0.0003 |
| **BMI** | 41.23 | 39.89 | 55.02 | 27.74 | 25.18 | 31.46 | 0.0313 |

Abbreviation. BMI: body mass index

**
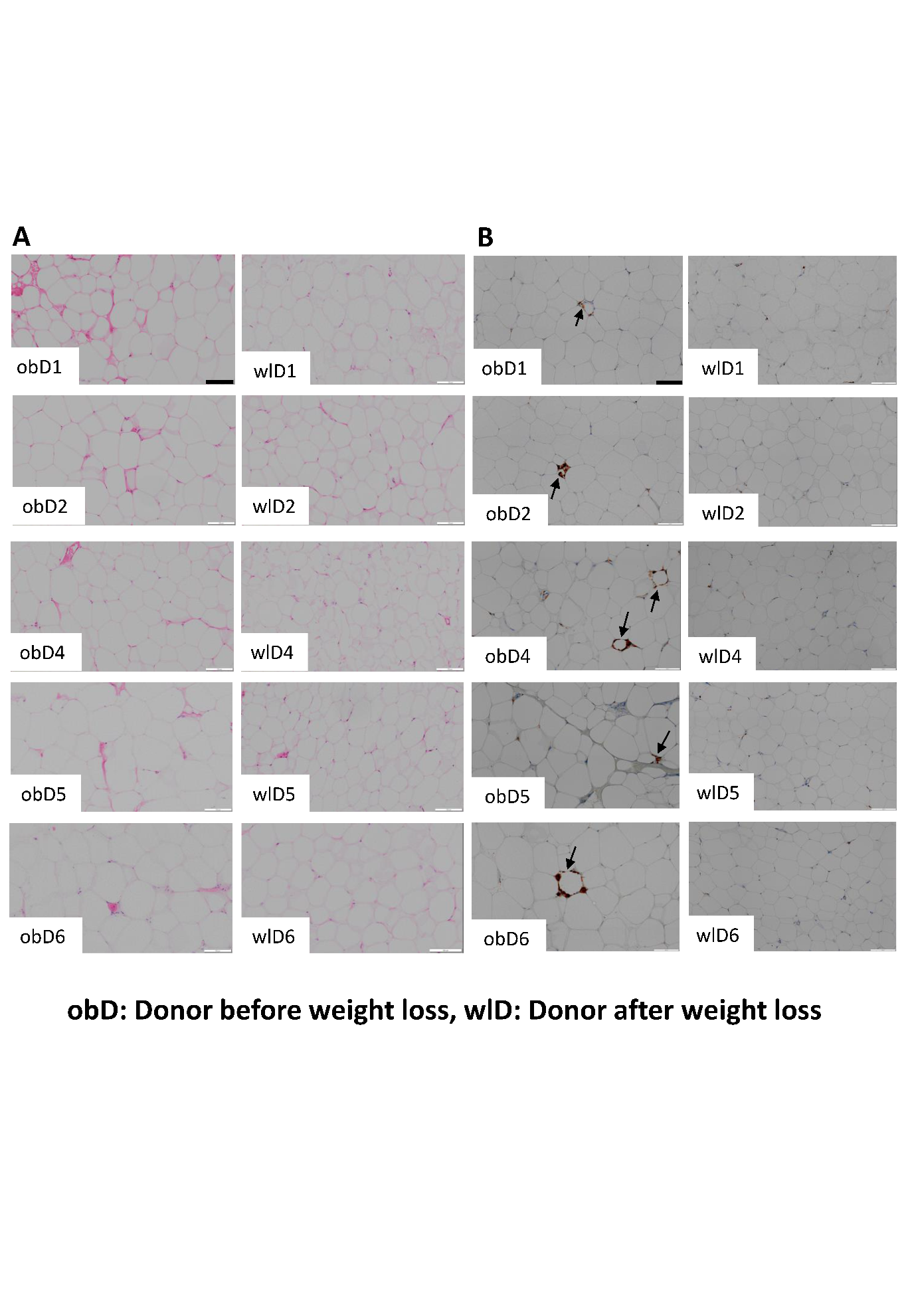
**

**Additional Figure 2. The effect of weight loss on adipocyte area and crown-like structures (CLS).** n=6. D) Adipocyte area in µm before and after weight-loss, A) Representative HE histological samples before and after the weight-loss from other five donors. B) Representative CD68 positive chromogenic histological samples before and after the weight-loss from other five donors. Arrows demonstrating the accumulation of (brown) macrophage CLS around the adipocyte. obD: Donor before weight loss, wlD: Donor after weight loss.


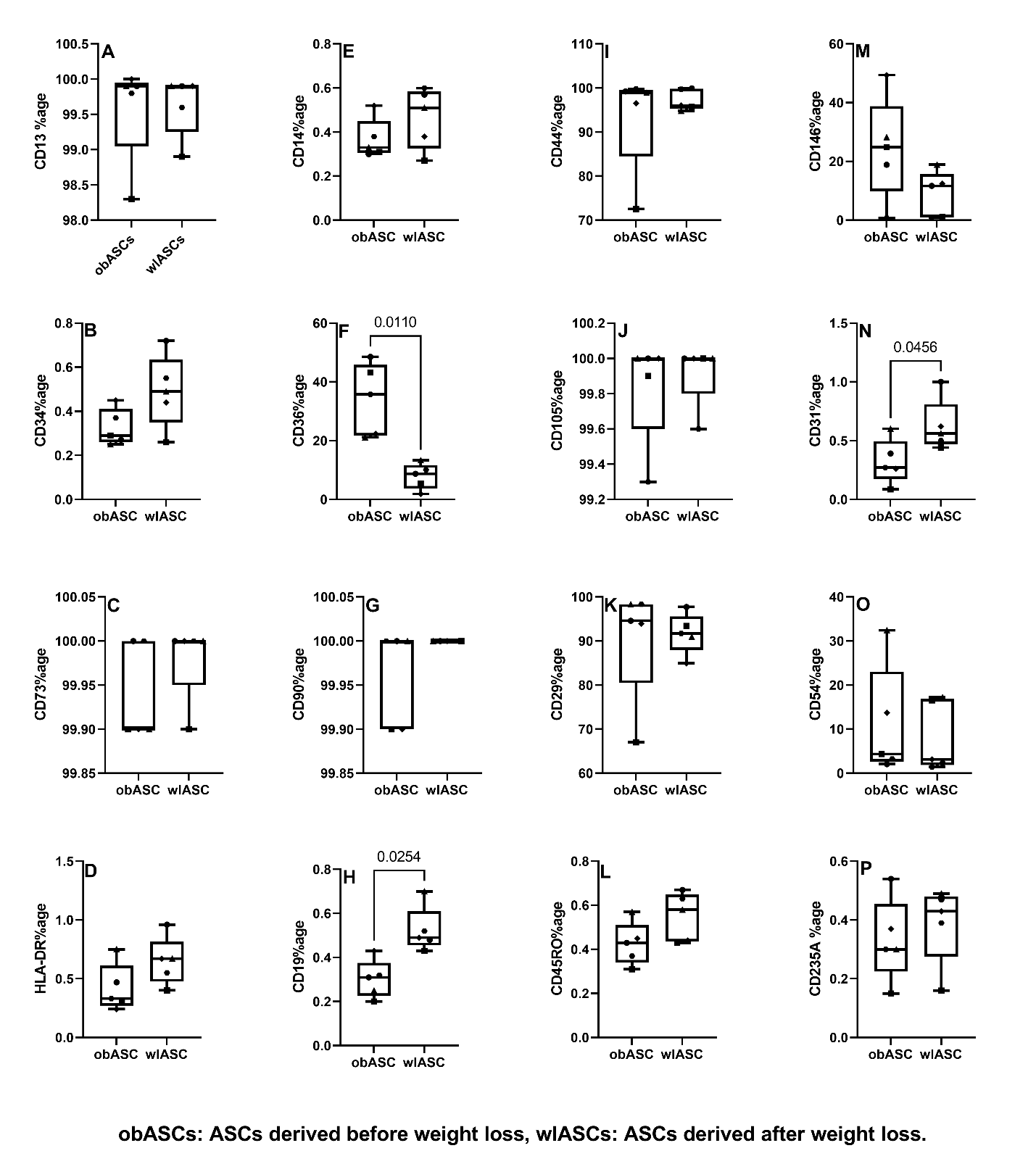


**Additional Figure 3. Comparison of different surface marker expressions between obASCs and wlASCs**. n=5. Values from the percentage of positive cells. obASCs: ASCs derived before weight loss, wlASCs: ASCs derived after weight loss. A) CD13, B) CD34, C) CD73, D) HLA-DR, E) CD14, F) CD36, G) CD90, H) CD19, I) CD44, J) CD105, K) CD29, L) CD45RO, M) CD146, N) CD31, O) CD54, P) CD235A. The paired t test was used for ASC surface marker expression. p values < 0.05 were considered significant. The data are presented as the minimum to maximum values.


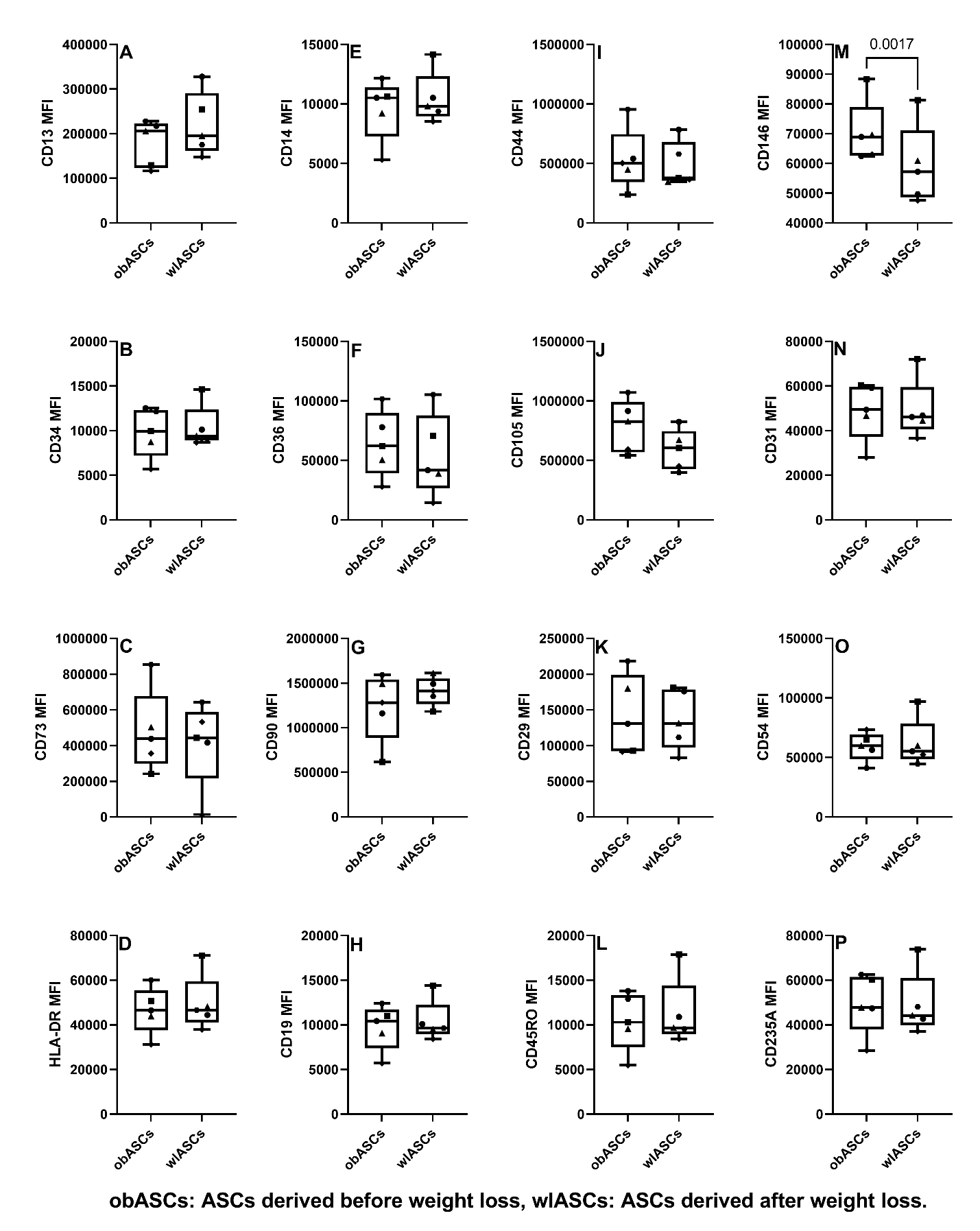


**Additional Figure 4. Comparison of different surface marker expressions between obASCs and wlASCs.** n=4. Values from the MFI of positive cells. MFI: median florescence intensity; obASCs: ASCs derived before weight loss, wlASCs: ASCs derived after weight loss. A) CD13, B) CD34, C) CD73, D) HLA-DR, E) CD14, F) CD36, G) CD90, H) CD19, I) CD44, J) CD105, K) CD29, L) CD45RO, M) CD146, N) CD31, O) CD54, P) CD235A. The paired t test was used for ASC surface marker expression. p values < 0.05 were considered significant. The data are presented as the minimum to maximum values.


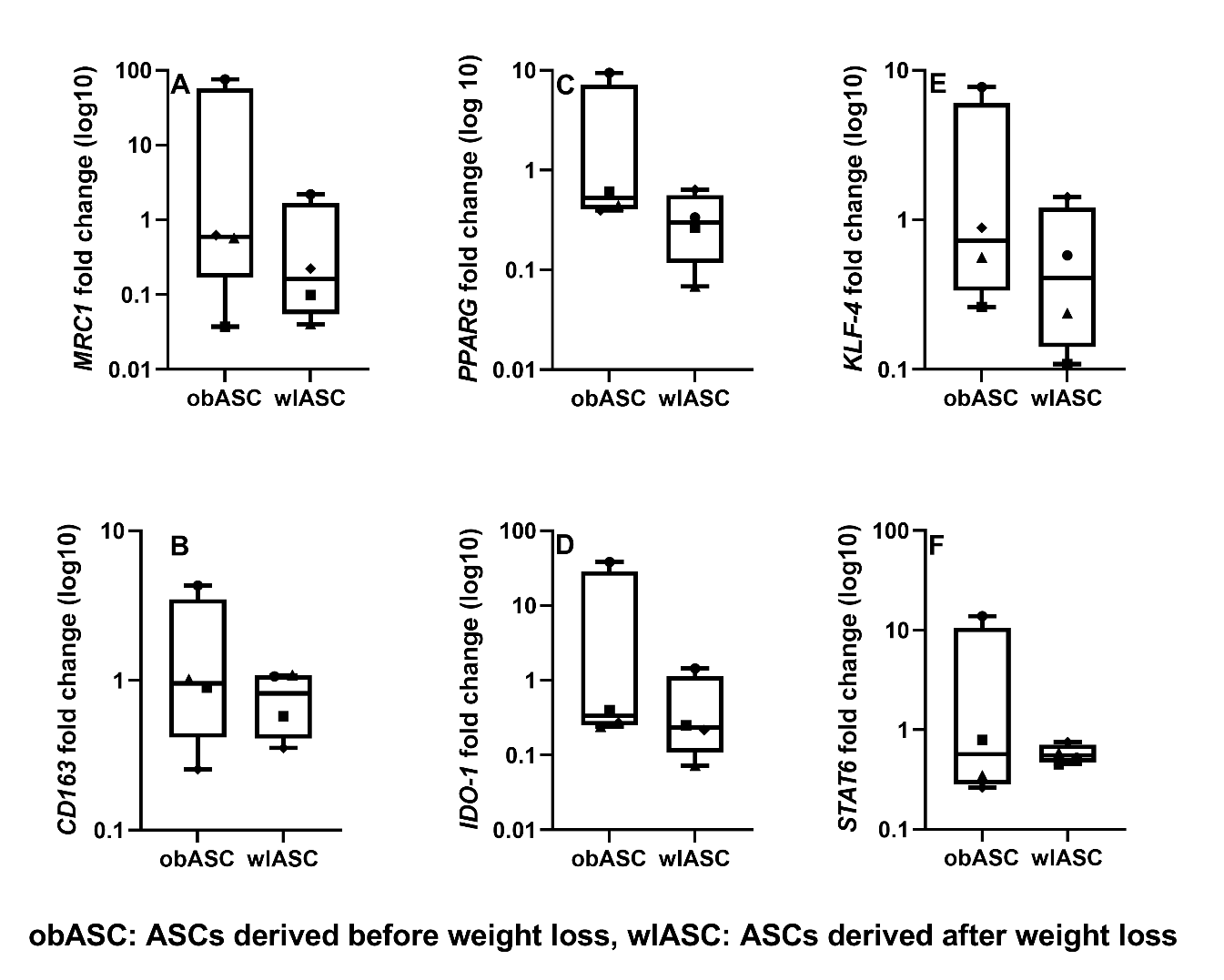


**Additional Figure 5. Comparison of gene expression between obASCs and wlASCs.** n=4. Box plots from fold change values and statistical analysis from the delta CT values. obASCs: ASCs derived before weight loss, wlASCs: ASCs derived after weight loss. A) *MRC1*, B) *CD163*, C) *PPAR-ɣ*, D) *IDO-1*, E) *KLF-4,* and F) *STAT6*. The Wilcoxon test was used for paired samples for gene expression. p values < 0.05 were considered significant. The data are presented as the minimum to maximum values.


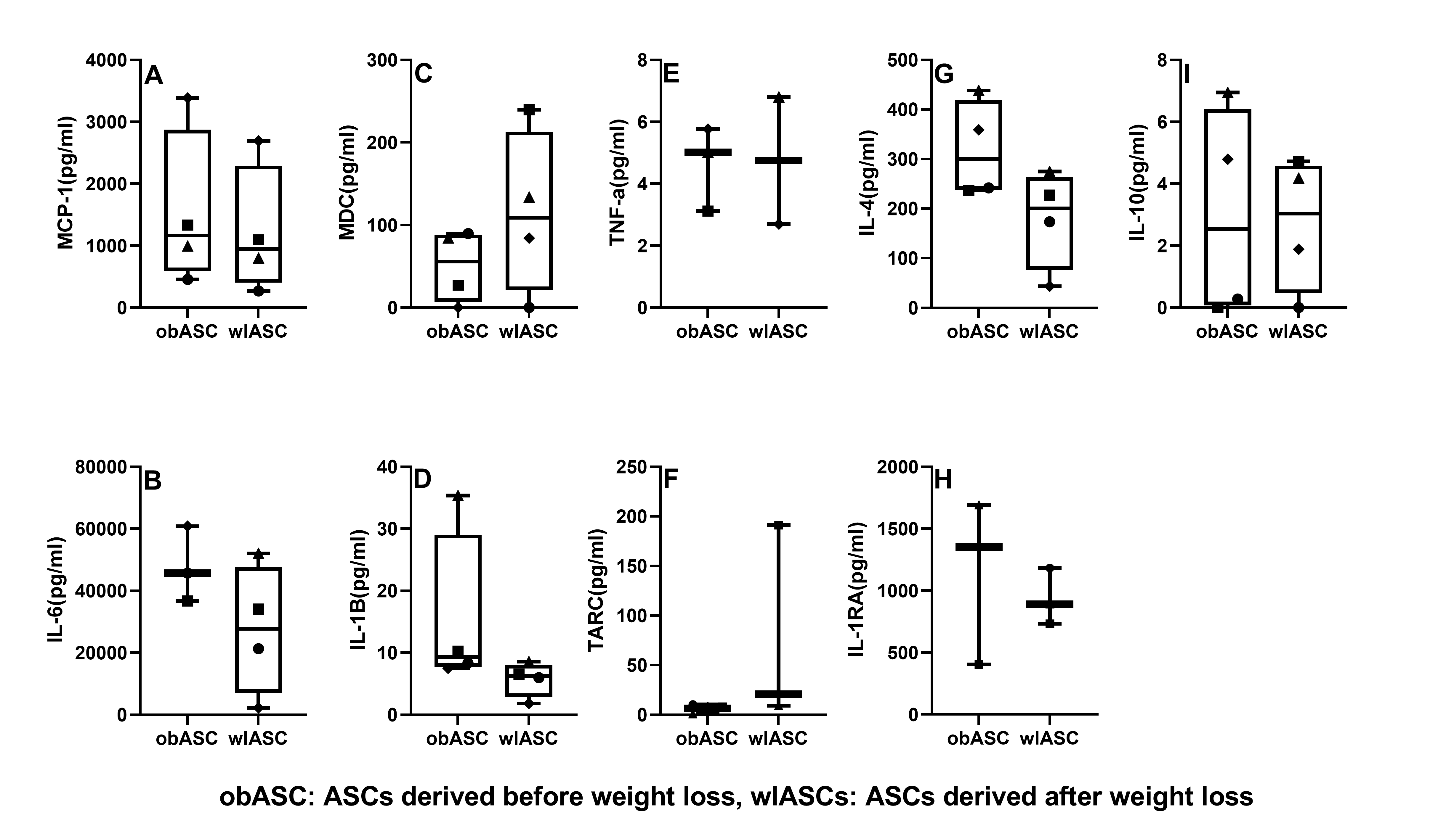


**Additional Figure 6. Comparison of cytokine secretion between obASCs and wlASCs.** n=4. obASCs: ASCs derived before weight loss, wlASCs: ASCs derived after weight loss. A) MCP-1, B) IL-6, C) MDC, D) IL-1β, E) TNF-α, F) TARC, G) IL-4, H) IL-1RA, and I) IL-10. The paired t test was used for cytokine secretion. p values < 0.05 were considered significant. The data are presented as the minimum to maximum values.

**Multipotent Differentiation Capacity of obASCs and wlASCs**

All obASCs (n=5) and wlASCs (n=5) lines had multipotential differentiation capacity and they differentiated towards osteogenic and chondrogenic lineages (Additional Figure 7A-B). On the other hand, decreased adipogenic differentiation was observed after the weight loss (Additional Figure 7C).


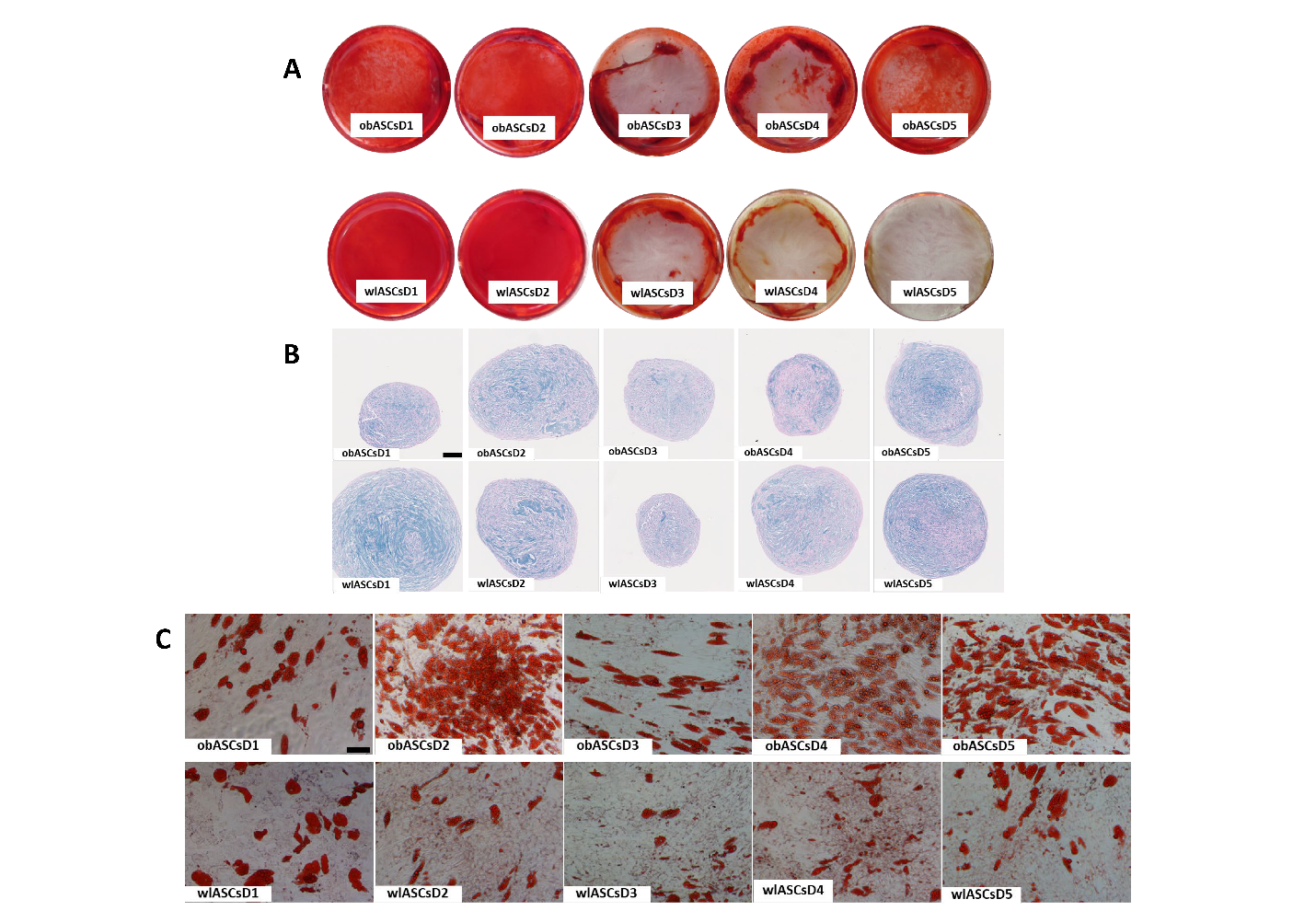


**Additional Figure 7. Multipotent differentiation capacity of ASCs before and after the weight loss.** n=5. A. Osteogenic differentiation, B. Chondrogenic differentiation, and C. Adipogenic differentiation of ASCs at passage 4 or 5. Scale bar 100µm. obASCsD: ASCs obtained before weight loss, wlASCsD: ASCs obtained after the weight loss. D refers to the donor number.

**Increased Proliferation after Weight loss**

Proliferation capacity of obASCs (n=5) and wlASC (n=5) were measured at day 7, 9 and 11. Increased while non-significant proliferation rate (Additional Figure 8) was observed after the weight loss.


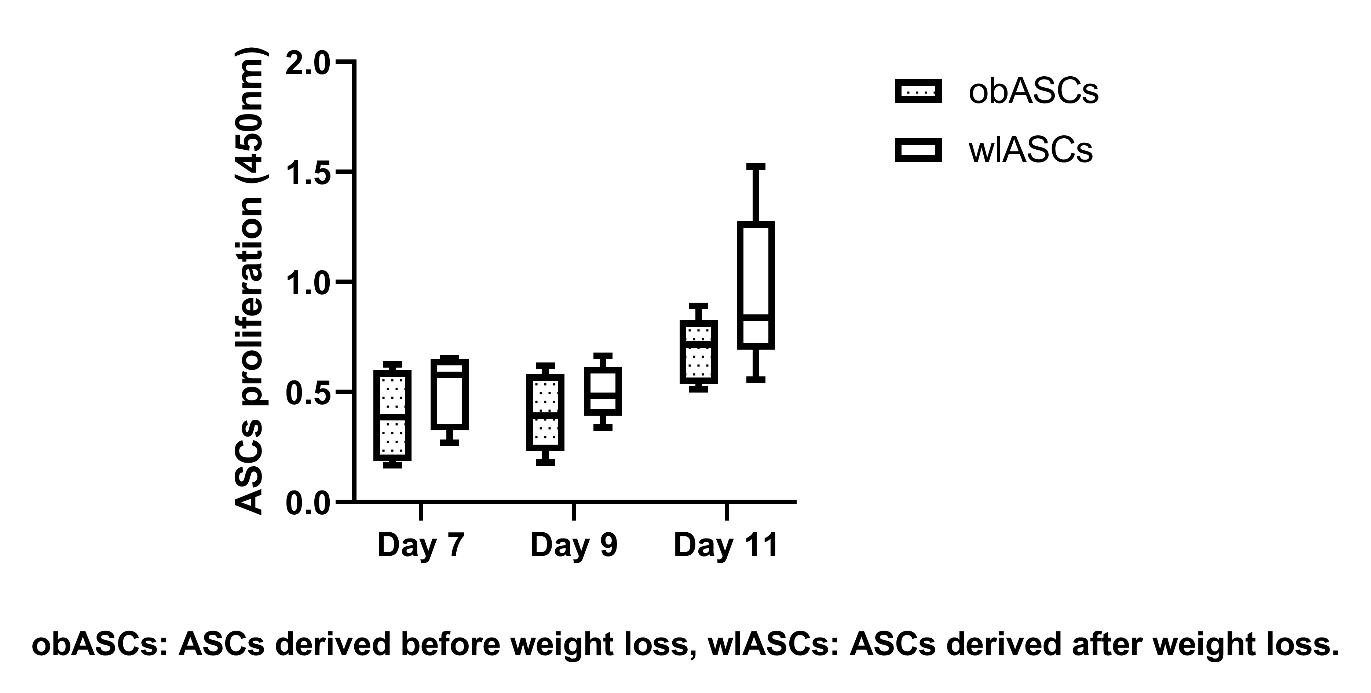


**Additional Figure 8. Proliferation capacity of ASCs before and after the weight loss.** n=5. Proliferation of ASCs measured at day 7, 9 and 11. Optical density measured at wavelength 450nm. obASCs: ASCs obtained before weight loss, wlASCs: ASCs obtained after the weight loss. The multiple paired t tests were used for ASC proliferation. p values < 0.05 were considered significant. The data are presented as the minimum to maximum values.

**Cytochemical Staining**

After macrophage activation, samples were fixed (4% PFA; Sigma-Aldrich) and permeabilized (0.1% triton-x-100; Sigma-Aldrich). The samples were then blocked (1% Bovine serum albumin; BSA; Sigma-Aldrich), stained with phalloidin (1:800 Phalloidin-Tetramethyl rhodamine B isothiocyanate; Sigma-Aldrich in 1% BSA), and counterstained with DAPI (1:2000 4’,6-diamidino-2-phenylindole dihydrochloride; Sigma-Aldrich in PBS), followed by some rinse [3]. Morphological characteristics of macrophages in monocultures were observed with Olympus IX51 (Olympus Corporation of the Americas, PA) after 6 days of culture. Images were processed with ImageJ.

**Phenotypic Characterization of Macrophage**

Pro-, and anti-inflammatory macrophages (M1, and M2 respectively), were polarized from frozen peripheral blood mononuclear cells (PBMCs). The cell morphology of macrophages was observed after thawing, four and six days of culture (Additional Figure 9). On the sixth day of culture after polarization, we studied differentiated morphologies such as, M1 appeared as round and M2 appeared as spindle as previously reported by Suzuki et al and Waldo et al [4], [5] (Additional Figure 9).

**
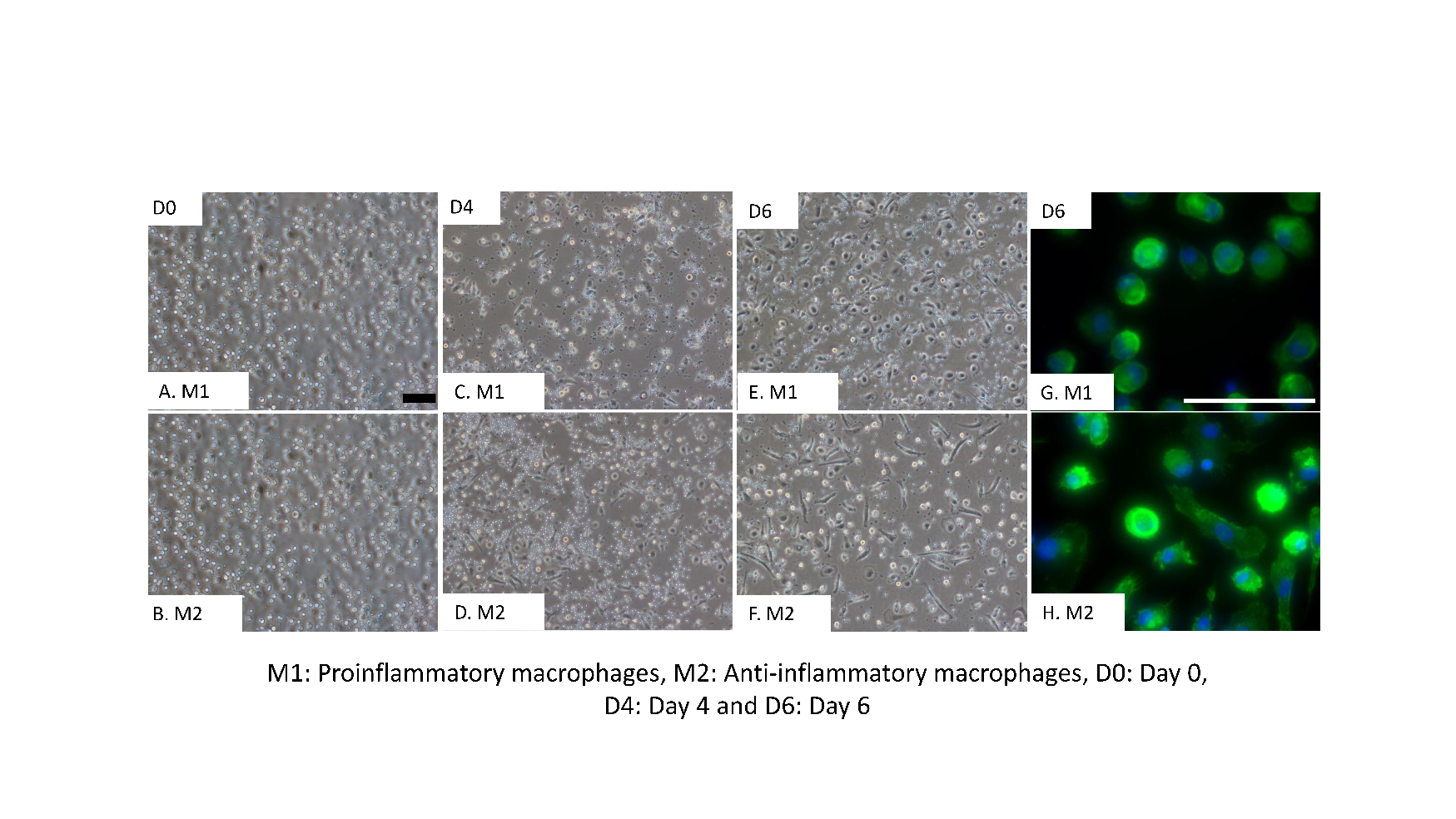
**

**Additional Figure 9. Morphology of different macrophages after 6 days culture in 10% FBS medium.** Light microscope images: A) M1, B) M2 at day 0. C) M1, D) M2 at day 4. E) M1, F) M2 and Florescence microscope images: G) M1, H) M2 at day 6. Scale 100µm. Blue shows nucleus stained with DAPI, and green shows cytoskeleton stained with phalloidin. M1: proinflammatory macrophages and M2: anti-inflammatory macrophages.

Furthermore, the polarization of different macrophages was also confirmed with flow cytometry (n=5). The macrophages were selected based on their size and granularity in the FlowJo analysis (Additional Figure 10-11)


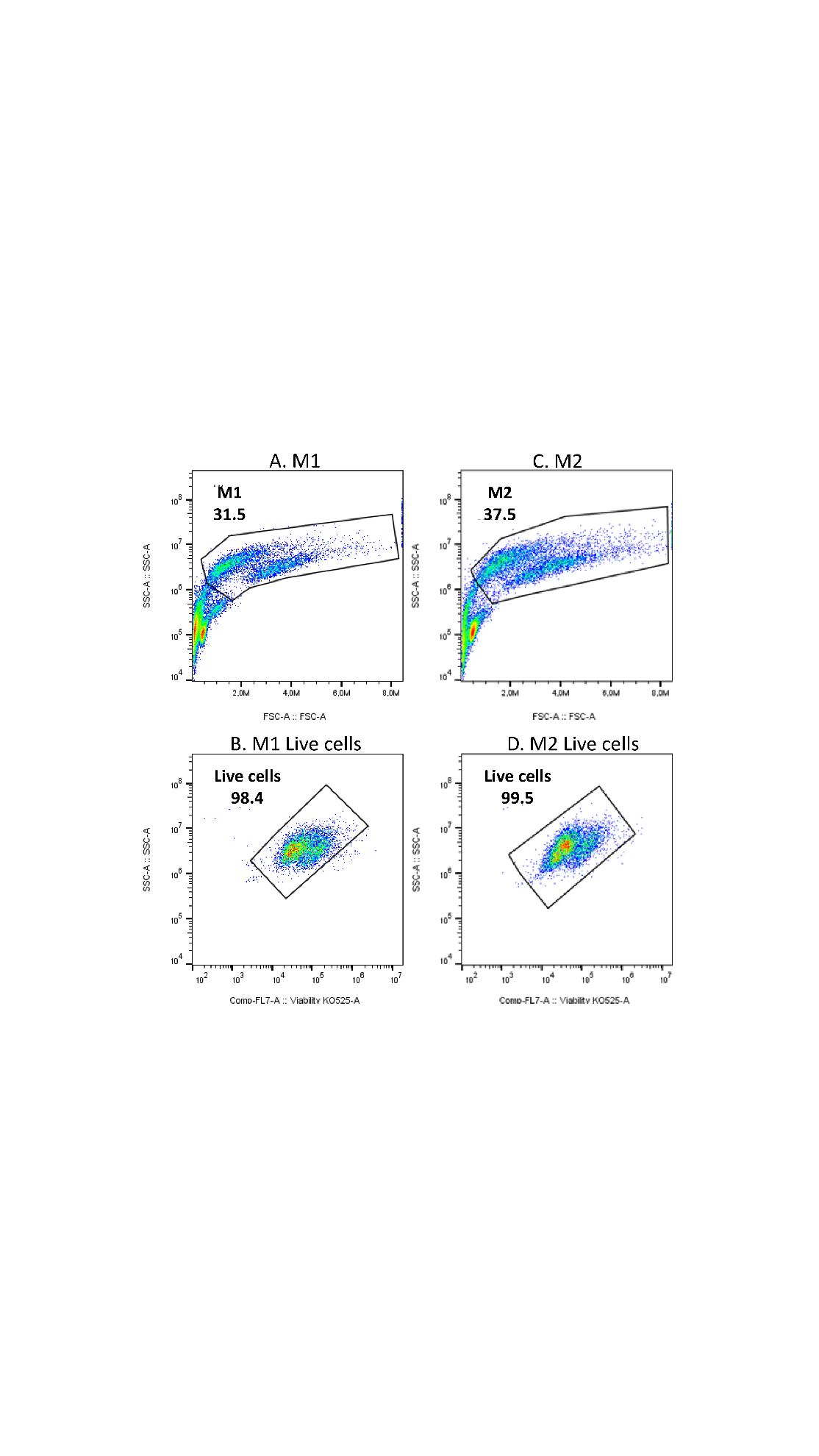


**Additional Figure 10. Representative gating images of M1, and M2 type macrophages from one experiment.** A) selection of M1, B) Live M1 cells, C) selection of M2, D) Live M2 cells. M1: Proinflammatory macrophages, M2: Anti-inflammatory macrophages.


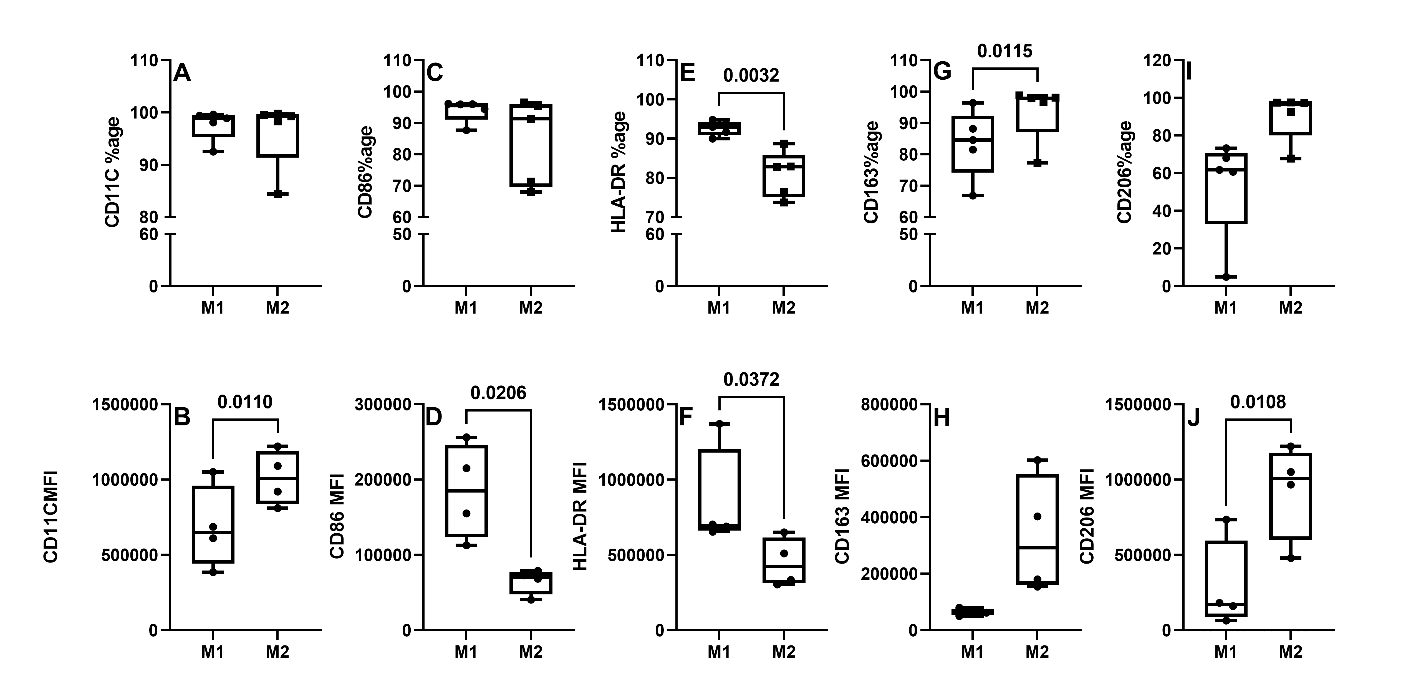


**Additional Figure 11.** **Surface markers of M1 and M2 type macrophages.** A; C; E; G; I: Percentage of positive cells, n=5. B; D; F; H; J: MFI of positive cells, n=4. A) CD11C%, B) CD11C MFI, C) CD86%, D) CD86 MFI, E) HLA-DR%, F) HLA-DR MFI, G) CD163%, H) CD163 MFI, I) CD206% and J) CD206 MFI. CD11C, CD86, HLA-DR: Human leukocyte antigen– DR isotype, CD163, CD206. MFI: median fluorescence intensity. The paired t test was used for macrophage surface marker analysis. p values < 0.05 were considered significant. The data are presented as the minimum to maximum values.


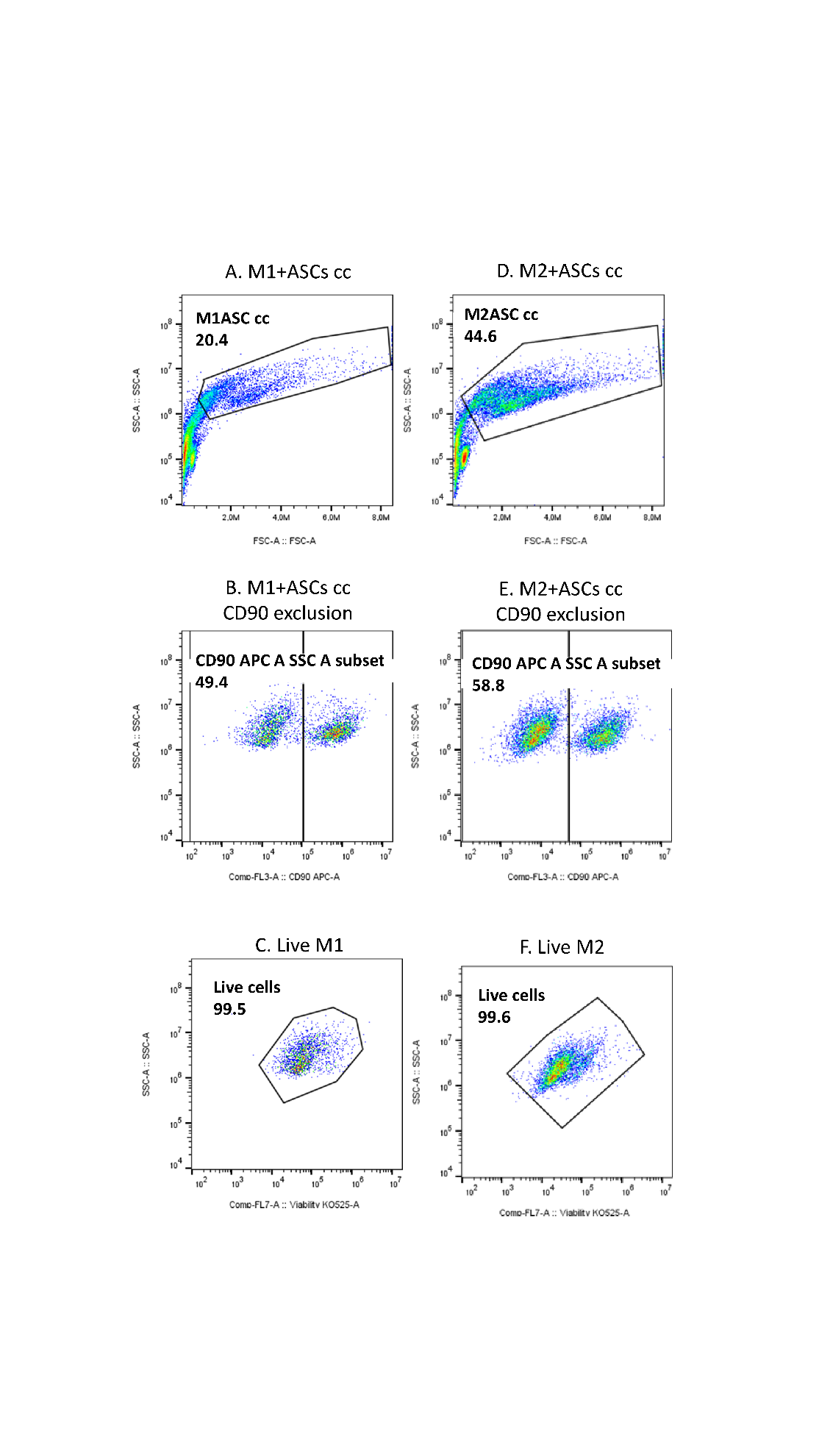


**Additional Figure 12. Representative gating images of M1 and M2 type macrophages with ASC cocultures from one donor.** A) selection of M1+ASCs coculture, B) Exclusion of ASCs from M1 coculture, C) Live M1 cells, D) selection of M2+ASCs coculture, E) Exclusion of ASCs from M2 coculture, F) Live M2 cells. M1: Proinflammatory macrophages, M2: Anti-inflammatory macrophages. ASCs cc: Adipose stem cell cocultures. CD90.


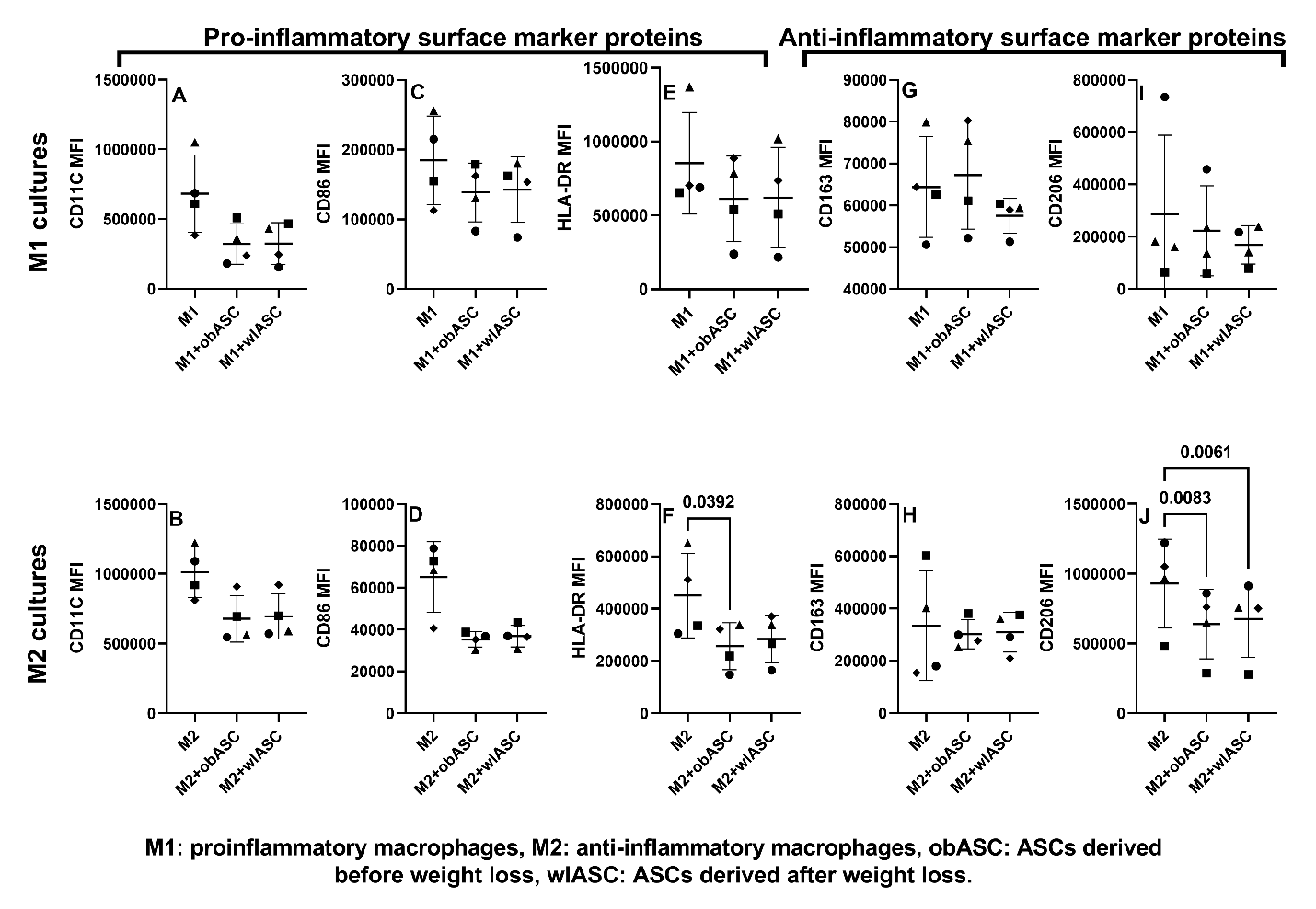


**Additional Figure 13. MFI of CD markers in M1, and M2 cells in monoculture and coculture with obASCs and wlASCs.** n=4. A) CD11C, C) CD86, E) HLA-DR, G) CD163, I) CD206 in M1 cultures. B) CD11C, D) CD86, F) HLA-DR, H) CD163, J) CD206 in M2 cultures. MFI: median fluorescence intensity, obASCs: ASCs derived before weight loss, wlASCs: ASCs derived after weight loss. For RM ANOVA, p values < 0.05 were considered significant. The data are presented as the means and SDs.


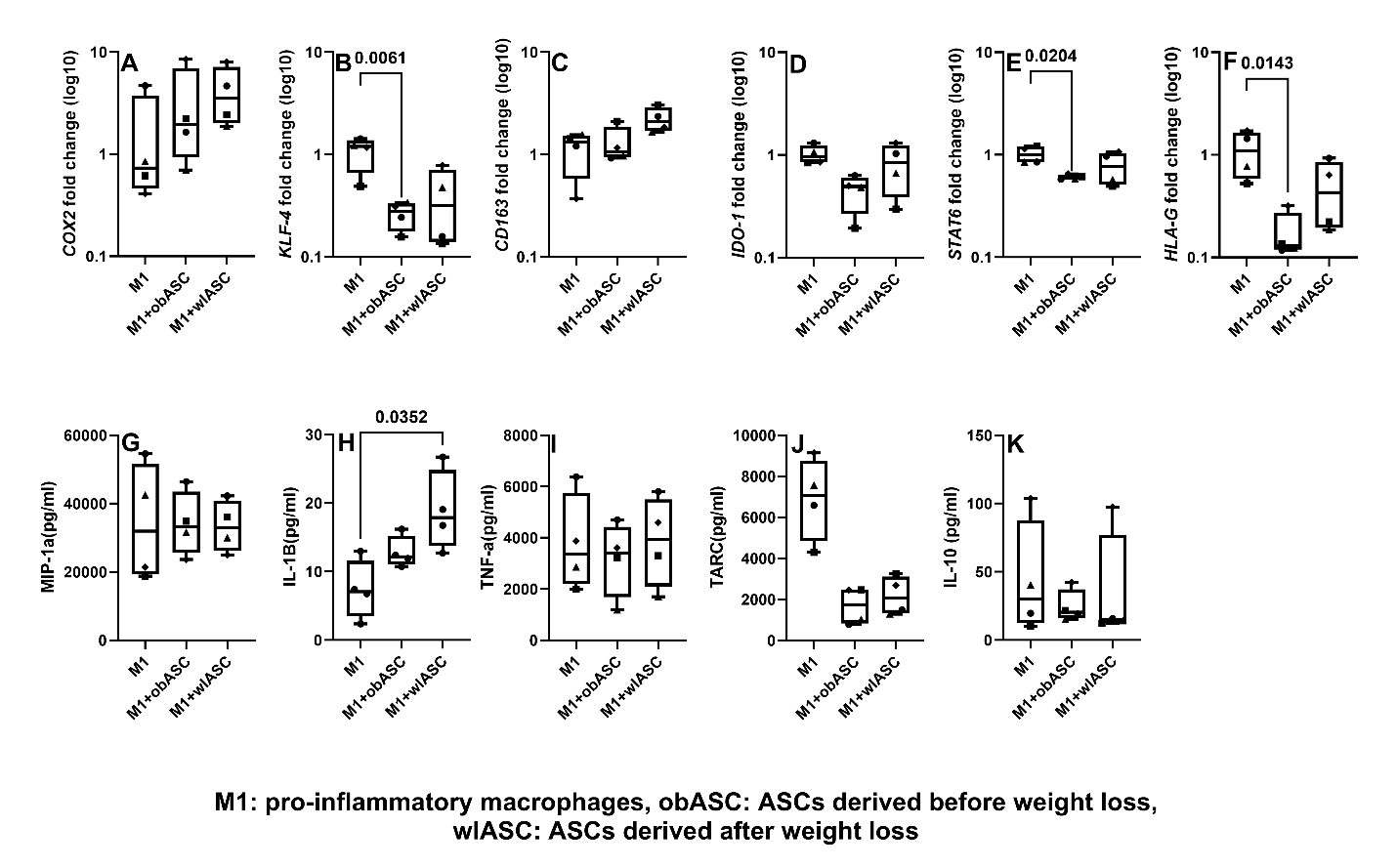


**Additional Figure 14. Comparison of gene expression and cytokine secretion between mono- and cocultures of M1 with obASCs and wlASCs.** n=4. A-F: Gene expression in M1 mono-/cocultures. G-K: Cytokine secretion in M1 mono-/cocultures. A) *COX2*, B) *KLF-4*, C) *CD163*, D) *IDO-1*, E) *STAT6*, F) *HLA-G*. G) MIP-1a, H) IL-1β, I) TNF-α, J) TARC, K) IL-10. obASCs: ASCs derived before weight loss and wlASCs: ASCs derived after weight loss. For RM ANOVA, p values < 0.05 were considered significant. The data are presented as the minimum to maximum values.


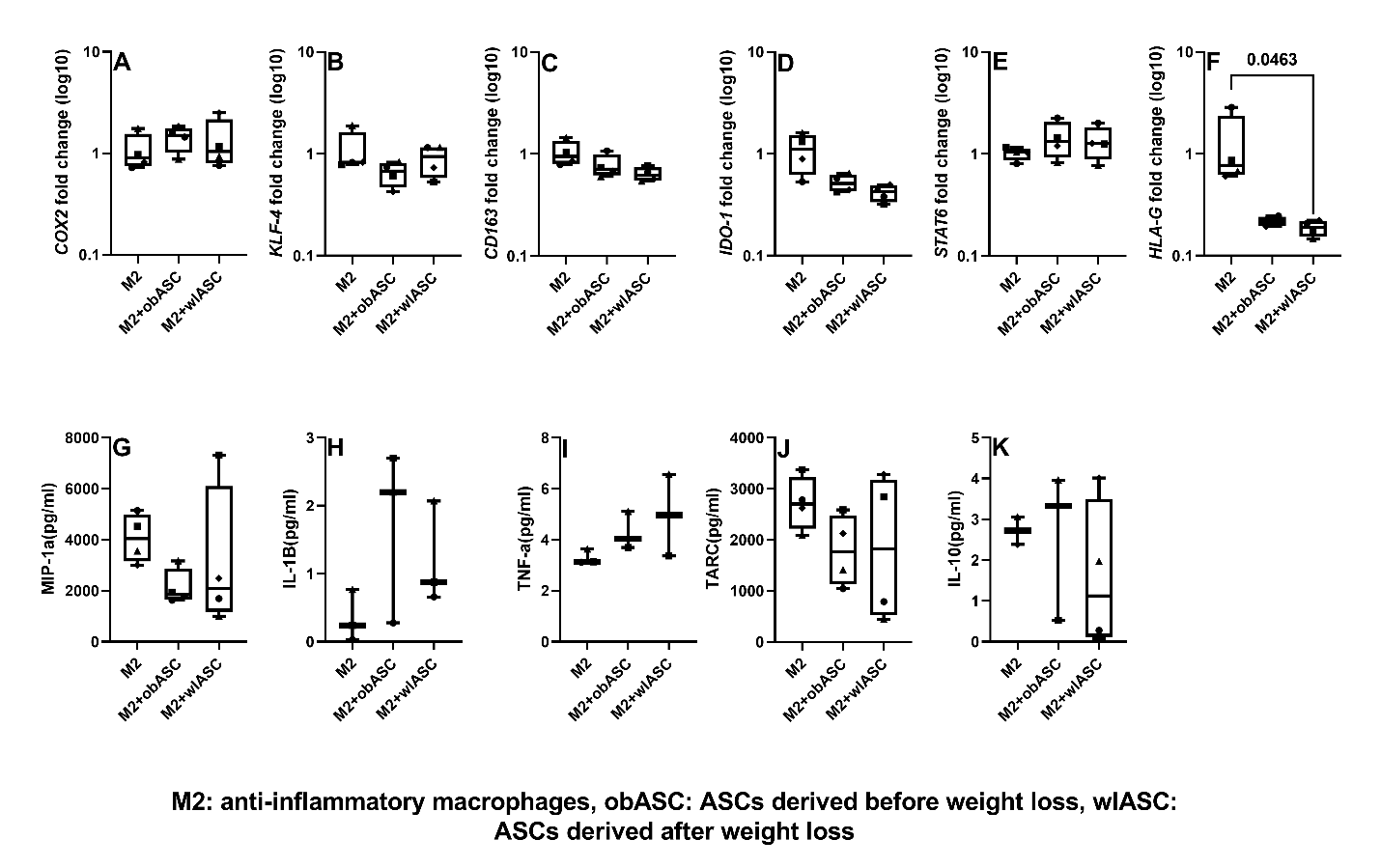


**Additional Figure 15. Comparison of gene expression and cytokine secretion between mono- and cocultures of M2 with obASCs and wlASCs.** n=4. A-F: Gene expression in M1 mono-/cocultures. G-K: Cytokine secretion in M2 mono-/cocultures. A) *COX2*, B) *KLF-4*, C) *CD163*, D) *IDO-1*, E) *STAT6*, F) *HLA-G*. G) MIP-1a, H) IL-1β, I) TNF-α, J) TARC, K) IL-10. obASCs: ASCs derived before weight loss and wlASCs: ASCs derived after weight loss. For RM ANOVA, p values < 0.05 were considered significant. The data are presented as the minimum to maximum values.

**REFERENCES**

1. Ojansivu, M., Vanhatupa, S., Björkvik, L., Häkkänen, H., Kellomäki, M., Autio, R., Ihalainen, J. A., Hupa, L., & Miettinen, S. (2015). Bioactive glass ions as strong enhancers of osteogenic differentiation in human adipose stem cells. Acta biomaterialia, 21, 190–203. https://doi.org/10.1016/j.actbio.2015.04.017
2. Mahmoud, M., Juntunen, M., Adnan, A., Kummola, L., Junttila, I. S., Kelloniemi, M., Tyrväinen, T., Huhtala, H., Abd El Fattah, A. I., Amr, K., El Erian, A. M., Patrikoski, M., & Miettinen, S. (2023). Immunomodulatory Functions of Adipose Mesenchymal Stromal/Stem Cell Derived From Donors With Type 2 Diabetes and Obesity on CD4 T Cells. *Stem cells (Dayton, Ohio)*, *41*(5), 505–519. https://doi.org/10.1093/stmcls/sxad021
3. Hyväri, L., Vanhatupa, S., Halonen, H. T., Kääriäinen, M., & Miettinen, S. (2020). Myocardin-Related Transcription Factor A (MRTF-A) Regulates the Balance between Adipogenesis and Osteogenesis of Human Adipose Stem Cells. Stem cells international, 2020, 8853541. https://doi.org/10.1155/2020/8853541
4. Waldo, S. W., Li, Y., Buono, C., Zhao, B., Billings, E. M., Chang, J., & Kruth, H. S. (2008). Heterogeneity of human macrophages in culture and in atherosclerotic plaques. *The American journal of pathology*, *172*(4), 1112–1126. https://doi.org/10.2353/ajpath.2008.070513.
5. Suzuki, H., Hisamatsu, T., Chiba, S., Mori, K., Kitazume, M. T., Shimamura, K., Nakamoto, N., Matsuoka, K., Ebinuma, H., Naganuma, M., & Kanai, T. (2016). Glycolytic pathway affects differentiation of human monocytes to regulatory macrophages. *Immunology letters*, *176*, 18–27. https://doi.org/10.1016/j.imlet.2016.05.009.
